# Recent and Promising Strategies for Robust, Sustainable, and Cost‐Friendly Production of Yogurt With Superior Quality

**DOI:** 10.1111/1541-4337.70587

**Published:** 2026-08-08

**Authors:** Shuangqing Zhao, Belay Tilahun Tadesse, Christian Solem

**Affiliations:** ^1^ National Food Institute Technical University of Denmark Lyngby Denmark

**Keywords:** β‐galactosidase, bioprotective culture, lactic acid bacteria, post‐acidification, yogurt fermentation

## Abstract

Yogurt is one of the most technologically mature fermented dairy products on the market, traditionally prepared using the two lactic acid bacteria (LAB) *Streptococcus* *thermophilus* and *Lactobacillus delbrueckii* subsp. *bulgaricus*. Global demand for yogurt continues to rise, driven by consumer interest in healthy, high‐protein diets with as few additives as possible. New lactose‐free variants and sugar‐reduced yogurts appeal to large consumer segments that either have poor lactose tolerance or focus on a healthy lifestyle. Despite developments in yogurt manufacturing technologies and yogurt starter cultures, some persistent challenges associated with yogurt remain, for example, post‐acidification problems, texture instability, and microbial spoilage, all of which shorten shelf life and contribute to food waste. Lactose‐free products, which often have reduced sugar content, are still considered niche and, as such, are sold at a premium, which deters many consumers from buying them and likely compels them to choose plant‐based yogurt analogs, which are often marketed as more sustainable. The current trend toward increased use of direct‐vat‐set (DVS) cultures, which increases production costs and thus prices at the retailer, could likewise have this effect. In this review, we provide a comprehensive and industry‐oriented analysis of existing and emerging strategies for achieving robust, sustainable, and cost‐effective production of yogurt. We address industrial applicability, limitations, regulatory considerations, and future development potential. We argue that there is a current trend toward the use of more natural, bio‐driven solutions for improving the properties of yogurt.

## 1 Introduction

Yogurt is one of the most widely studied fermented dairy products and is appreciated worldwide. During yogurt fermentation, the accumulation of lactic acid decreases the pH, which drives casein micelles to aggregate and form a continuous gel network (Chandan and Kilara 2013). The resulting yogurt is a mildly acidic, palatable dairy product containing abundant live lactic acid bacteria (LAB), and its high content of easily digested high‐quality proteins, lipids, carbohydrates, minerals (including calcium), and vitamins gives yogurt a high nutritional value (Xu et al. 2025). All these properties explain why the market for yogurt is expanding. In 2025, the size of the global yogurt market was $50.32 billion, and it is expected to reach $53.4 billion by 2026 and $91.14 billion by 2030 (Compound Annual Growth Rate [CAGR] ≈ 6.12%; Towards FnB 2026). With a 43% share of global value sales and the highest growth rates, Asia leads the world in yogurt consumption, mainly driven by China and Japan, where the demand is growing faster than in all other markets, including Brazil and the United States (Innova Market Insight 2025).

Despite the favorable prospects for the global yogurt market, the dairy industry still faces several challenges. To maintain yogurt texture, exogenous stabilizers such as gelatin and pectin are commonly added to preserve quality throughout shelf life; however, their use interferes with the clean‐label status that an increasing number of consumers desire (Eze et al. 2021). Post‐acidification during storage of yogurt largely impairs its sensory quality and shelf‐life stability. This is due to the persistence of metabolic activity of *Lactobacillus delbrueckii subsp. bulgaricus* (Zhou et al. 2025). In parallel, yogurt spoilage also causes massive food waste, for example, an estimated 42,000 tonnes of yogurt is wasted annually in the United Kingdom alone (Ridler 2022), which directly conflicts with sustainability objectives and international efforts to reduce food loss and waste. Use of chemical preservatives is an efficient way to control yogurt spoilage, but chemicals such as potassium sorbate have been reported to greatly reduce the count of *Streptococcus thermophilus* in yogurt (Cuttance et al. 2025). While most of the artificial preservatives are generally regarded as safe, some have been linked to toxic or even carcinogenic effects. This has led to a rise in consumer demand for minimally processed, synthetic‐additive‐free foods, which, in turn, has increased the interest in natural preservation methods (Dwivedi et al. 2017). A good example of the latter is microbial bio‐preservation, where food microorganisms that naturally inhibit spoilage microorganisms are harnessed to extend shelf life (Argyri et al. 2022).

Another challenge that yogurt is facing is its high cost, and here expensive starter cultures make a considerable contribution; the value of raw milk is currently around €0.43 /kg in the EU (MilkUA.info 2026), where the starter culture costs around €0.05 /kg fermented milk, that is, 10% of the raw milk price (Hjemmeriet 2026). Yogurt direct‐vat‐set (DVS) starter cultures are expensive to manufacture as their production involves costly industrial‐scale fermentation, concentration, freeze‐drying, and cold‐chain distribution. Furthermore, DVS cultures are highly reliant on a specific supplier (Majumder et al. 2026).

Yet another challenge is the inclusion of excessive amounts of sugar in yogurt in order to improve its taste. Added sugar reduces the health benefits of yogurt and consequently consumer confidence by questioning the credibility of health claims associated with yogurt products (Wan et al. 2021). Taken together, these limitations highlight the need for innovative and natural solutions that harness microorganisms to improve yogurt texture, flavor, and shelf life that can fulfill health‐oriented expectations of the consumers.

Being able to account for the use of sustainable technologies in production has become a license to operate for dairies. Surveys carried out to determine purchasing patterns for yogurt have revealed that health benefits are a primary driver (48%), exceeding taste as the main motivation for purchasing yogurt (Innova Market Insights 2025). This has forced dairies to produce yogurts that are safe, nutritious, and sensorially attractive, in addition to meeting clean‐label and sustainability requirements. Upstream suppliers of raw materials and ingredients, that is, milk, starter cultures, and functional additives, are forced to cope with higher costs due to production logistics uncertainty (OECD/FAO 2023). On the other hand, downstream retailers are seeking consistent product quality, extended shelf life, and affordable prices due to the evolving, high‐variety nature of their markets (Bianchi and Venturi 2025). For the US dairy sector, nearly 70% of companies reported margins in 2025 as flat or getting worse versus 66% in 2024 and 58% in 2023, highlighting a steady erosion rather than a short‐term squeeze. Europe is facing similar strains, with 57% of companies reporting flat or declining margins (Dariy Processing 2026). These data also highlight the urgency for dairy companies to undergo a sustainable development transformation.

Concurrently, the dominating position of yogurt is being seriously threatened by surging interest in plant‐based analogs. Manufacturers of plant‐based solutions generally claim that their product is more sustainable, caters to dietary needs (e.g., vegans and allergy‐prone individuals), and contributes a variety of bioactive compounds, all of which resonate well with health‐ and sustainability‐oriented consumers. Legumes (e.g., soybeans, peas, and mung beans), cereals and pseudocereals (e.g., oats, millet, and quinoa), nuts (e.g., almonds and walnuts), and coconut milk are currently the most popular raw materials used in the production of plant‐based yogurt analogs (Montemurro et al. 2021). However, compared with many plant‐based alternatives, regular yogurt generally provides proteins with higher digestibility and a more balanced indispensable amino acid profile. Based on the digestible indispensable amino acid score (DIAAS) system proposed by FAO/World Health Organization (WHO), dairy proteins typically exhibit higher nutritional quality than many plant‐derived proteins, which are often limited in one or more essential amino acids (Herreman et al. 2020). Consequently, yogurt offers nutritional advantages in terms of protein quality and amino acid availability. For those subsisting on a vegan diet or a diet with a restricted intake of animal‐derived foods, calcium and vitamin B12 must be sourced from other sources, as they are often low or absent in plants (Melina et al. 2016). Despite the nutritional gap, plant‐based solutions are constantly being improved due to research and development efforts, for example, by fortifying them with proteins, vitamins, and minerals and by the addition of probiotics (Hossain et al. 2025; Montemurro et al. 2021). Plant‐based yogurt analogs are seen as more environmentally friendly by consumers due to their often lower carbon footprint and less resource‐demanding production (Carlsson Kanyama et al. 2021). Thus, plant‐based yogurt alternatives are considered substitutes for traditional yogurt by many consumers. Based on these considerations, it is time to take a fresh look at how yogurt can be produced, focusing on the clean‐label status, cost efficiency, and technological innovation to keep pace with new forces in the market.

This review summarizes recent indications, underrepresented advancements, and recent regulations governing yogurt products in yogurt production, including strategies to minimize post‐acidification during storage, microbial bio‐preservation approaches to limit the use of chemical preservatives, emerging methods to generate bulk starters to reduce yogurt costs, and alternative solutions for producing lactose‐free yogurt with enhanced natural sweetness, as well as a comparison of yogurt regulatory differences between countries. Together, these innovations illustrate a shift toward more natural, biologically driven solutions for manufacturing yogurt with improved properties.

## 2 Traditional and Recent Developments in Yogurt Production

Yogurt has a long history of consumption, dating back to ancient times. Although the precise circumstances of its discovery remain uncertain, the Mesopotamians around 5000 BC have often been credited as potential early pioneers of yogurt (Chandan and Kilara 2013). Yogurt remained a relatively unfamiliar product in Europe until the early 20th century. Nobel Prize‐winning immunologist Ilya Metchnikov established a connection between the longer lifespans of certain groups in Bulgaria, Russia, France, and the United States and their consumption of fermented milk products, and it was indicated that these products could deter the growth of harmful bacteria by acidifying the digestive tract (Anadón et al. 2021). In the late 1920s, large‐scale yogurt production began with milder yogurt varieties flavored with fruit. Broader popularity was achieved in the 1960s, thanks to advancements in Swiss flavor and fruit inclusion, as well as the French development of a stable, creamy, stirred yogurt (Schmid and McGee 1989).

**FIGURE 1:**
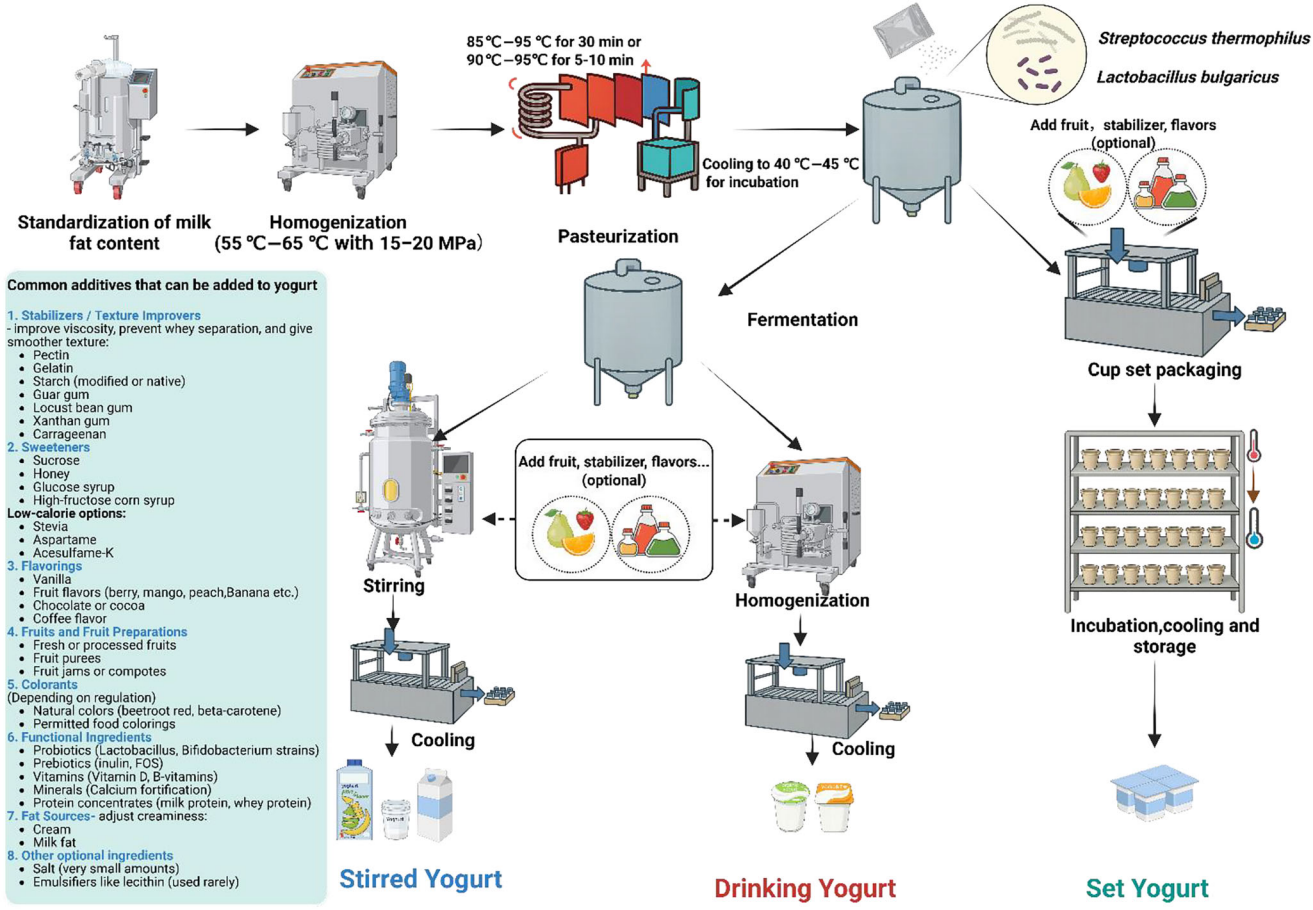
Classification of yogurt production processes and applicable additives.

Yogurt is one of the most important food products, fermented from milk by LAB and contains many nutrients. As shown in Figure 1, there are three main categories of yogurt: set yogurt, stirred yogurt, and drinking yogurt (Le Ba et al. 2025). Set yogurt ferments directly in its container and is often available in plain or flavored varieties. In contrast, stirred yogurt undergoes fermentation, is blended, homogenized (even liquefied for drinking yogurts) and packaged. Drinking yogurt is obtained by further homogenizing or diluting stirred yogurt, sometimes adding stabilizers for a smoother texture and more fluid, uniform consistency (Sobhay et al. 2019).

Standard yogurt contains only two types of bacterial species, namely, *L. bulgaricus* and *S. thermophilus*. During yogurt production, milk is fermented at 42°C to 45°C, and each bacterium stimulates the growth of the other. Collectively, due to their classic proto‐cooperative interaction, they acidify milk more rapidly than they are capable of individually. *S. thermophilus* first consumes oxygen and produces formate and CO_2_, which stimulates the growth of *L. bulgaricus*. On the other hand, *L. bulgaricus* exhibits marked proteolytic activity, liberating peptides and amino acids that support the growth of *S. thermophilus*. This metabolic interaction improves lactose catabolism and increases the acid production rate during yogurt fermentation (Wu et al. 2025). Yogurt is now manufactured in various amounts and flavors with different fat contents. Additionally, yogurt exhibits unique regional characteristics and traditional production methods that vary based on geographical areas and cultural backgrounds. For example, Greek yogurt undergoes straining to separate the whey, resulting in a thicker, creamier texture than regular yogurt. It is known for its high protein content and is often used in both sweet and savory dishes. However, during Greek yogurt production, 100 units of milk are converted into 33 units of yogurt and 67 units of acid whey, which is challenging to dispose of (Britt 2017; Karela et al. 2025). To maintain and improve the quality, stability, and nutritional value of yogurt, additives play a crucial role. As shown in Figure 1, common functional additives include stabilizers such as pectin, gelatin, and modified starch, which enhance viscosity and prevent syneresis by reinforcing the protein–water network (Y. Zhang et al. 2025). Sweeteners such as sucrose and honey are commonly added to yogurt to enhance its sweetness (Sarkar and Chandra 2019). Flavor additives, including natural vanilla, fruit preparations, and aromatic compounds, enhance sensory appeal without compromising fermentation performance (Das et al. 2025). Functional ingredients, such as probiotics (*Lactobacillus*, *Bifidobacterium*), prebiotics (e.g., inulin, fructooligosaccharides), vitamins, minerals, and protein concentrates, help tailor yogurt to specific nutritional profiles (Kaewarsar et al. 2023; D. Li et al. 2023; S. Zhang et al. 2025). Emerging trends also highlight the use of bioactive components such as antioxidants, plant extracts, and peptides to create value‐added functional yogurts (X. Wang et al. 2025). Altogether, these additives enable manufacturers to modulate texture, flavor, health properties, and shelf stability, making them essential tools in modern yogurt formulation.

The core value proposition of yogurt is transitioning from nutrition to a more advanced health role, increasingly on gut health—but also health as a whole. The combination of probiotics and prebiotics has emerged as a focus for product development, with the aim of improving gut health and balance to support digestive function and a healthy immune system. Consumers these days are also inclined toward yogurts enriched with novel proteins, as they opt for fitness and healthy foods rich in nutrients. Due to the presence of nutritional bioactives such as lactoferrin, vitamins, and minerals, yogurt is also a more acceptable vehicle for daily nutritional fortification (Gomes Da Costa et al. 2020). Similarly, there is an increasing demand from consumers for clean‐label yogurt with fewer or no food additives, simplified ingredients lists, that are low in sugar or sugar‐free (Maruyama et al. 2021). Taken together, these trends illustrate the progression of yogurt innovation: function, nutritional enhancement, and minimal formulation.

**FIGURE 2:**
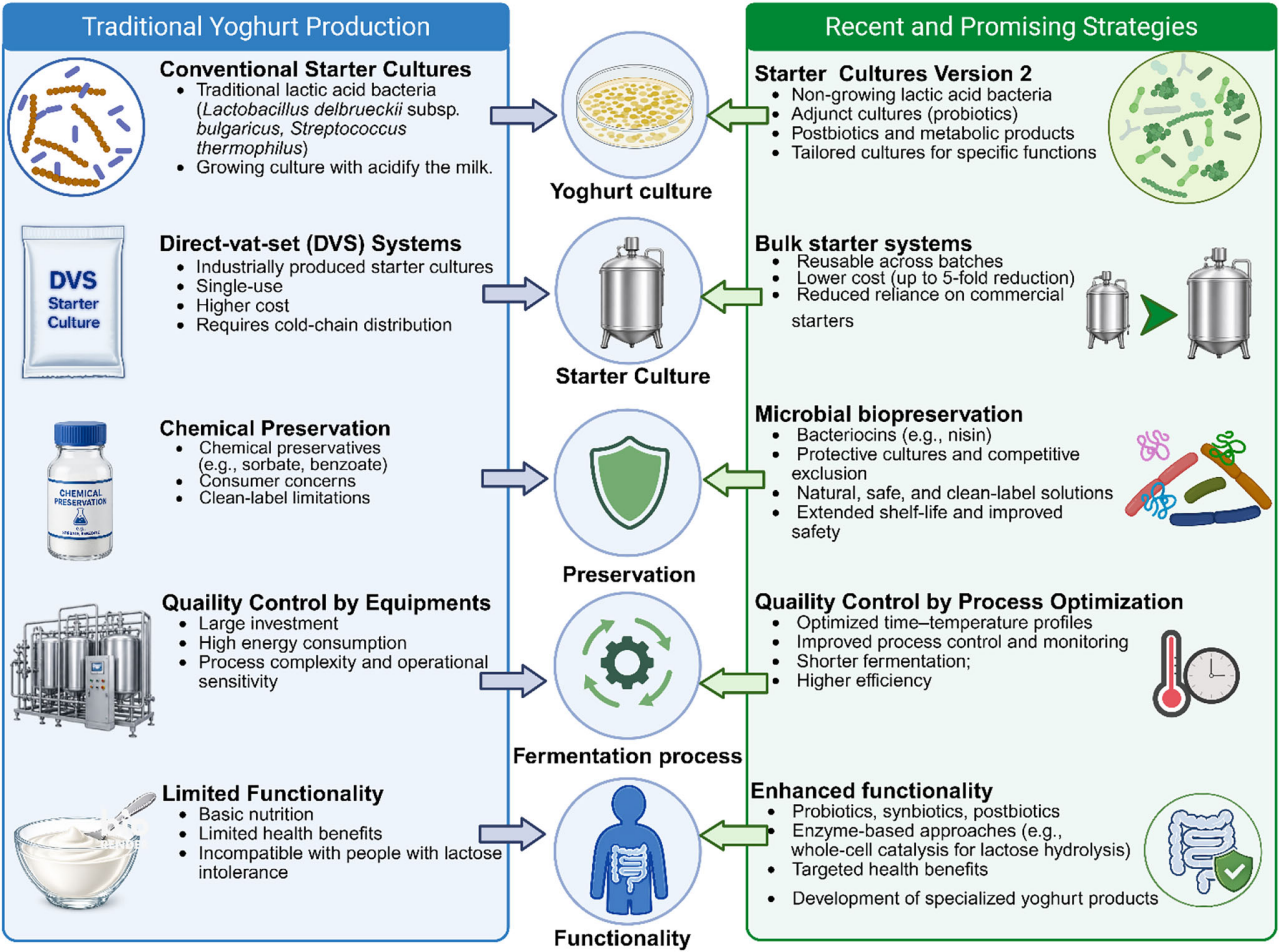
Evolution of yogurt production: traditional approaches and recent developments.

Figure 2 further illustrates this shift, summarizing the transition from traditional yogurt production to more advanced, sustainable production strategies. These latest developments include the use of improved or next‐generation starter cultures, the adoption of bulk starter systems to reduce costs, microbial bio‐preservation methods for clean‐label preservation, and process optimization to improve fermentation efficiency. A major challenge is the reluctance of dairies to adopt novel technology, which contributes to making the transition from traditional to more sustainable production methods more difficult. By utilizing postbiotics and enzyme technologies, yogurt can be transformed into lactose‐free yogurt, suitable for lactose‐intolerant consumers. Simultaneously, microbial metabolites offer health benefits. Microbial bio‐preservation is increasingly replacing chemical preservatives, meeting consumer demand for “clean label” products and better compliance with yogurt regulations across different countries and regions, thereby contributing to expanding the market. At the same time, changing the fermentation process and optimizing yogurt fermentation parameters will reduce production costs from another perspective, transforming quality control, previously reliant on heavy equipment, into an efficient, low‐energy process management. We believe this comprehensive and multifaceted evolution in yogurt production will transform dairy companies from simply producing nutritious foods into high‐value‐added, highly efficient, and environmentally sustainable health enterprises.

For the yogurt industry in general, the emphasis of the short‐term research will be on further enhancing the industrial robustness and scale‐up of these emerging technologies. It contributes to improving starter culture performance, preventing post‐acidification, building bio‐preservation systems supporting clean label status, and reducing sugar and lactose levels without compromising sensory quality. In addition, upstream processing should be improved to enhance process reproducibility and compliance, as well as economic feasibility under industrial production conditions. Finally, this area of research could evolve toward microbiome‐targeted functional yogurt products in the long run, coupled with AI‐assisted fermentation control and starter culture systems tailored to precise nutritional/sensory/sustainability targets. Additionally, for the next‐generation yogurt products, incorporating sustainable production methods, low‐carbon processing technologies, and probiotic dairy production approaches will likely be important.

## 3 Microbial Strategies for Yogurt Shelf‐Life Extension

Yogurt spoilage poses a significant economic and quality challenge for both consumers and yogurt manufacturers. Besides contributing to food waste, a limited shelf life also interferes with the export of yogurt to foreign markets due to time‐consuming logistics, thereby reducing retailer shelf life (Mercier et al. 2019). Among spoilage microorganisms, yeasts and molds are the most common types. Yeasts are generally more tolerant of acidic environments than most bacteria and can grow at pH values as low as 3.0, which explains why they are commonly found as spoilage organisms in acidic foods such as yogurt (Ikalafeng 2001). It is reported that yeast survives under low pH conditions due to special modifications of its cell wall (Kapteyn et al. 2001). At low pH, the yeast cell wall is organized in a finely controlled and fine‐tuned manner that is governed by the cell wall integrity signaling pathway. This pathway transduces wall stress signals from the cell surface to the Rho1 GTPase, which triggers a physiologic response through a variety of effectors (Levin 2011). Acid tolerance in yeast can be achieved by altering sterol composition and reducing iron absorption, which for *Saccharomyces cerevisiae* results in acid tolerance to a pH as low as 2.8 (Fletcher et al. 2017).

Inadequately sealed or post‐contaminated yogurt products can also be spoiled by molds, more specifically *Penicillium*, *Cladosporium* spp. and *Aspergillus* spp., that may lead to visible mold growth, off‐flavor production, texture damage, and potentially build‐up of mycotoxins, which pose a serious health threat (Erfani et al. 2024). So, controlling yeast and mold spoilage in yogurt remains a critical priority when seeking strategies to prevent or limit fungal growth and development in dairy products.

### 3.1 Industrial Strategies for Controlling Yogurt Spoilage and Extending Shelf Life

Industrial strategies for extending the shelf life of yogurt can be broadly categorized into three main types: (i) preventative measures aimed at reducing contamination during processing and packaging; (ii) physicochemical and processing interventions to inhibit microbial growth; and (iii) biological methods such as microbial bio‐preservation. These strategies differ in their mechanisms of action, industrial applicability, and impact on product quality, and are typically used in combination in practice to achieve the best shelf‐life extension effect.

Preventive measures to reduce the risk of yogurt contamination during processing include aseptic packaging, air filtration, and ensuring a sanitary work environment, in accordance with hygiene regulations, including HACCP (Hazard Analysis and Critical Control Points; Mishra et al. 2024). Control strategies, by contrast, rely on retarding, preventing, or killing fungi through methods such as chemical preservatives and post‐process treatments, including modified atmosphere packaging, cooling treatment, heat treatment, high‐pressure processing, and through the use of bioprotective cultures (Garnier et al. 2017). Among them, microbial strategies have been recognized as a practical strategy for extending yogurt shelf life. Yogurt protective cultures are selected food bacteria that can be added to fermented dairy products and yield natural antifungal metabolites that limit the growth of spoilage yeasts and molds. Antifungal compounds are frequently formed during microbial metabolism of carbohydrates and amino acids and comprise organic acids such as lactic acid, acetic acid, and phenyllactic acid (PLA). Certain microorganisms produce specific antifungal peptides or cyclic compounds that can disrupt fungal cell membranes or interfere with cell metabolism, thereby inhibiting fungal growth (A. Liu et al. 2022). As shown in Table 1, a variety of representative commercial bioprotective cultures, mainly comprising *Lactobacillus* and *Propionibacterium*, are added together with yogurt starter culture for preservation purposes. Although the exact mechanisms by which they work remain unclear, current evidence indicates that their antifungal activity usually arises from a combination of metabolic and ecological interactions in the dairy matrix. Although these effects have been mainly described in dairy systems, similar antifungal mechanisms can also be found in other food matrices (Nasrollahzadeh et al. 2022; Nazareth et al. 2023). The principal mechanisms cited in the papers are production of weak organic acids, generation of various antifungal metabolites, and competition for key nutrients, especially manganese. The following subsections will elaborate on these mechanisms in further detail.

**TABLE 1:** Representative commercial bioprotective cultures applied in yogurt preservation.

Product name	Source	Function	Application	Manufacturer	Reference
FreshQ 2	*Lactobacillus rhamnosus*	Primarily produces CO_2_ and diacetyl due to citrate fermentation and does not produce lactic acid	Yogurt (set yogurt, stirred yogurt, Greek yogurt)	Novonesis	BHG (2026)
FreshQ 4	*Lactobacillus rhamnosus Lactobacillus paracasei*	Combinations of traditional lactic acid bacteria (LAB) that deliver inhibition of unwanted yeast and molds in fermented dairy products	Yogurt, fresh cheese, and sour cream	Novonesis	Chr Hansen (2021)
HOLDBAC YM‐XPM	*Lactobacillus paracasei Lactobacillus plantarum*	A blend of patented *Lactobacillus paracasei* and *Lactobacillus plantarum* that have been selected and designed for optimal yeast and mold inhibition while maintaining a milk‐acidic flavor	Yogurt and other fresh fermented dairy products	IFF	Dairy Connection (2026d)
HOLDBAC YM‐B	*Lactobacillus rhamnosus Propionibacterium freudenreichii* subsp. *shermanii*	Combined with acidifying cultures to generate by‐products such as L(+) lactic acid, acetic acid, and propionic acid, along with small amounts of other organic acids and diacetyl, thereby slowing yeast and mold growth	Fresh cheeses (mozzarella, feta, cottage cheese) and fermented‐milk products (yogurt, sour cream, quark)	IFF	Dairy Connection (2026b)
HOLDBAC YM‐C	*Lactobacillus paracasei Propionibacterium freudenreichii *subsp.* shermanii*	These cultures are combined with acidifying cultures to produce by‐products such as L(+) lactic acid, acetic acid, and propionic acid, along with small amounts of other organic acids and diacetyl, thereby inhibiting yeast and mold growth	High‐moisture cheeses such as fresh cheeses (mozzarella, feta, cottage cheese) and fermented‐milk products (yogurt, sour cream, quark)	IFF	Dairy Connection (2026c)
Delvo Guard 301	*Lactobacillus rhamnosus*	It contains cultures that are metabolically active specifically against yeast and molds in fermented food products.	For yogurt, added directly to milk or the fermentation mix without prior activation	DSM‐Firmenich	Dsm‐firmenich (2026a)
LyoPro TECT+	*Lactobacillus rhamnosus* and *Propionibacterium shermanii*	With slow acidification, this culture produces acetic and propionic acids, biologically suppressing the growth and activity of unwanted microorganisms such as heterofermentative *Leuconostoc*, enterococci, yeasts, and molds.	Cheese and yogurt	Codex Ing Biotech Ingredients	Codex‐ing (2026)
Lyofast LPR A	*Lactobacillus rhamnosus* and *Lactobacillus plantarum*	The culture develops a mild acidity and pleasant aroma through slow citrate fermentation in yogurt and also inhibits the growth of undesirable microorganisms.	Cheese (sprayed on surface) and yogurt (added to milk)	Sacco Srl.	Sacco (2021)
LYOFAST LRB	*Lactobacillus rhamnosus*	The culture develops a weak acidity and aroma from slow citrate fermentation. It is a protective culture inhibiting yeasts and molds.	Fermented milk products and cheese products as non‐starter culture LAB	Sacco Srl.	Sacco (2014)
LYOFAST LR 4 PD	Two selected strains of *Lactobacillus rhamnosus*	It is a protective culture that inhibits the growth of yeasts and molds. This product helps maintain the quality of the final food by naturally preventing spoilage and suppressing undesirable microorganisms.	Cheese (sprayed on the surface) and yogurt (added to milk)	Sacco Srl.	Dynamiko Food Ingredients (2026)

### 3.2 Production of Weak Organic Acids

During fermentation, protective *Lactobacillus* strains generate weak organic acids, primarily lactic and acetic acid, which reduce pH and create an unfavorable environment for yeasts and molds (Siedler et al. 2019). The propionic acid bacterium *Propionibacterium freudenreichii*, which can convert lactate into antifungal propionate, has also been applied in bioprotective cultures (Dank et al. 2021). Additionally, benzoic acid can be generated through the conversion of hippuric acid in milk by various LAB (Garmiene et al. 2010). One strain, *L. spicheri* O15 could produce 2.7 mg/L azelaic acid and 22 mg/L PLA (Le Lay et al. 2016). However, most of the above‐mentioned acids are usually produced by LAB in amounts that are lower than the minimum inhibitory concentration (Siedler et al. 2019).

To date, no microorganism with bioprotective properties has been found to produce an acid that fully inhibits spoilage microorganisms in yogurt. Some papers suggest that the undissociated forms of these acids can diffuse into fungal cells, altering intracellular pH and disrupting metabolic pathways (Sorathiya et al. 2025). Therefore, weak organic acids serve as complementary but not decisive spoilage‐limiting agents, pointing toward a possible crucial synergistic or multi‐factorial defense strategy in fermented dairy systems.

### 3.3 Production of Secondary Metabolites With Antifungal Activity

Besides organic acids, protective cultures are also reported to synthesize a range of natural antifungal compounds, including diacetyl (Shi and Knøchel 2021), fatty acids (Liang et al. 2022), reuterin (Pilote‐Fortin et al. 2021), reutericyclin (Gänzle et al. 2000), and several low‐molecular‐weight antifungal peptides such as a novel casein‐derived antifungal peptide (DMPIQAFLLY) produced by bioprotective *Lactobacillus* strains in sour cream. For the latter, certain bacteria produce specific proteinases and peptidases such as endo‐peptidase, di‐peptidase, and tripeptidase that hydrolyze milk proteins (e.g., casein) in a manner that releases these unique antifungal peptides (Anusha and Bindhu 2016; Kashung and Karuthapandian 2025). Although their maximum natural concentration (∼0.4 µM) is far below the ≥ 35 µM required for fungal inhibition, these peptides still slow down the growth of, for instance, *Debaryomyces hansenii*. This suggests that protein‐derived metabolites may contribute synergistically to LAB‐mediated bioprotection (McNair et al. 2018).

Other metabolites interfere with fungal membrane function, cell wall biosynthesis, and energy metabolism and have been shown to significantly delay visible spoilage in yogurt and other fermented milk products. An excellent review has thoroughly summarized the antifungal compounds produced by LAB and their underlying mechanisms (Crowley et al. 2013), and the reader is referred to this and other reviews. It should be mentioned that only a few antifungal LAB are commercially available. This paucity is probably due to the variation in antifungal activity of a given strain depending on a number of its physicochemical conditions, such as food processing and the capability of this strain to produce active metabolites in situ during food fermentation. Moreover, even when a strain can produce effective antifungal secondary metabolites, these compounds or other associated by‐products may negatively affect the sensory or textural quality of yogurt (Crowley et al. 2013), diacetyl being a good example. Diacetyl is a key aroma compound in yogurt and also exhibits antifungal activity against spoilage fungi. For example, *P. commune* 01,180,002 was completely inhibited in yogurt by diacetyl at 16 µg/mL at 16°C; however, at 25°C, complete inhibition required a higher concentration of 64 µg/mL (Shi and Knøchel 2021). The concentrations of diacetyl produced by co‐cultures of *L. plantarum* and *L. harbinensis* ranged from 3.3 to 13.4 mg/kg (Leyva Salas et al. 2017), which exceed the typical levels of diacetyl found in yogurt (0.2–3 mg/kg). While diacetyl contributes desirable buttery notes at low concentrations, the higher levels have a negative effect on the sensory quality of yogurt (Cheng 2010; Lima et al. 2026). Reuterin, produced by *Limosilactobacillus reuteri* from glycerol, exhibits potential as a biological preservative in dairy products. It is reported that when 18.25 mM reuterin is added to raw milk used for producing yogurt, it markedly alters yogurt properties, including color (turns pinkish red), interferes with acidification thereby elevating pH, decreases yogurt starter culture viability, and reacts with casein, leading to the conversion of casein monomers into oligomers (Sun et al. 2025; Vimont et al. 2019). This remains a significant challenge for both dairies and suppliers of bioprotective solutions.

### 3.4 Nutrient Competition Through Manganese Scavenging

Recent research has uncovered the role of nutrient competition, especially the depletion of essential micronutrients such as metal ions, in the antifungal activity of LAB. Several *Lactobacillus* species can inhibit spoilage yeasts and molds in fermented milk by depleting manganese (Mn^2^^+^), which is essential for fungal growth. This antifungal ability is driven by the highly expressed MntH1 manganese transporter, which enables rapid and exhaustive Mn^2^^+^ uptake from the dairy matrix. The expression of MntH1 is strongly strain‐dependent and influenced by species interactions and growth phase, and deletion of the gene abolishes manganese depletion and the associated bioprotective effect (Siedler et al. 2020). By taking up manganese, these bacteria reduce its availability for spoilage fungi. Since fungi require manganese for growth, this limits fungal growth. Use of these strains to prevent spoilage of yogurt or similar fermented milk products provides a compelling clean‐label option as opposed to artificial preservatives. Although manganese scavenging is an effective measure against *D. hansenii*, *S. cerevisiae*, and *Rhodotorula mucilaginosa*, it is less effective against *Candida fragicola* and *Torulaspora delbrueckii*. Although the use of manganese scavenging as a strategy to control growth of spoilage fungi is more recent, the phenomenon is well‐known; early studies in the 1940s already explored the mineral requirements of LAB, including K^+^, Na^+^, Mg^2^^+^, Mn^2^^+^, Fe^2^^+^, PO_4_^3−^, SO_4_^2−^, and Cl^−^. Nevertheless, we believe that revisiting these fundamental mineral dependencies may provide valuable insights for the rational design and development of next‐generation bioprotective cultures (MacLeod and Snell 1947).

To sum up, even though protective cultures are widely used in fermented milk products, their true bioprotective mechanisms remain only partially elucidated. Crucially, their efficacy is relatively dependent on spoilage yeast (or mold) species. It has been reported that when *L. rhamnosus* M.V8.6.2F (5 × 10^6^ CFU/mL) and *D. hansenii* (10^4^ yeast cells/mL) were co‐inoculated in milk, the growth of the yeasts was completely inhibited. However, no inhibitory effect was observed against *Kluyveromyces lactis* CLIB 196, whereas only slight growth retardation was observed for *K. marxianus* CLIB 282 and *Yarrowia lipolytica* LMSA 2.11.004, as compared to the control (Delavenne et al. 2013). Although these reported cultures can contribute to the prevention of fungal growth, they can also modify yogurt sensory characteristics, increasing sourness due to organic acid production, bitterness from proteolysis‐derived oligo‐peptides, and off‐flavors caused by the accumulation of volatile compounds like diacetyl and fatty acids (Aguirre‐Garcia et al. 2024; Ye et al. 2023). In practice, bioprotective cultures of this type generally only give a shelf‐life extension effect that is one to 2 weeks long, which may be helpful to support the export business of dairies. Thus, the bioprotective culture use requires a cost–benefit analysis that balances the additional cost of buying specific protective cultures with actual product losses and sensory effects on the yogurt. Another important issue is the post‐acidification, with pH values decreasing below the typical pH range (≈4.0–4.6) to below 3.85 during storage (Aryana and Olson 2017; Donkor et al. 2007), thereby negatively affecting texture and sensory quality. Thus, choosing bioprotective cultures with low acidification capacity or using them in combination with mildly acidifying starters is important for achieving high‐quality yogurt with an optimal shelf life.

Regarding bioprotective cultures, future studies will need to focus on increasing their industrial robustness and, at the same time, on ensuring strain specificity and compatibility with sensory properties under real production conditions. In the longer run, improvements in microbial ecology, metabolomics, and AI‐supported fermentation control should deliver precision‐targeted bioprotective systems with directed antifungal profiles coupled to less post‐acidification whilst being amenable to hosting a clean‐label yogurt production.

### 3.5 Biological Oxygen Scavengers—A New Approach to Inhibit Fungal Spoilage in Yogurt

In yogurt production, contamination with fungal spores from various sources in the dairy plant environment is likely. Spores can originate from the air, contaminated packaging materials, starter cultures, ingredients, or stabilizing agents. Spoilage fungi include genera such as *Penicillium*, *Aspergillus*, *Rhizopus*, *Fusarium*, and *Mucor*. Oxygen is important for fungal cellular metabolism, as it serves as a terminal electron acceptor in mitochondrial respiration for energy generation and is needed for the biosynthesis of sterols, unsaturated fatty acids, NAD, and porphyrins (Grahl et al. 2012). Since most fungi are obligate aerobes, that is, require oxygen to grow, their growth can be prevented merely by eliminating oxygen (Garnier et al. 2017). Furthermore, oxidative basidiomycetous yeasts including *Cryptococcus*, *Rhodotorula*, and *Sporidiobolus*, together with most facultative anaerobic spoilage yeasts, generally grow more rapidly under aerobic conditions (Hernández et al. 2018; Ledenbach and Marshall 2009). Limitations in production equipment at some dairy factories can result in residual oxygen in yogurt packaging, allowing spoilage fungi to grow (Agriculture Institute 2023). This issue significantly compromises the shelf life and safety of yogurt.

One interesting approach for eliminating oxygen involves the use of the bacterium *P. freudenreichii*, which can utilize O_2_ when it oxidizes lactate to CO_2_ (Dank et al. 2021). As shown in Table 1, this may partially explain the antifungal effect of this bacterium, which is included in bioprotective cultures such as HOLDBAC YM‐B and HOLDBAC YM‐B; besides its production of antifungal propionic acid, this microorganism depletes O_2_ in sealed yogurt containers, thereby inhibiting the growth of strict aerobic fungi and, in this way, prolongs shelf life (Garnier et al. 2017).

**FIGURE 3:**
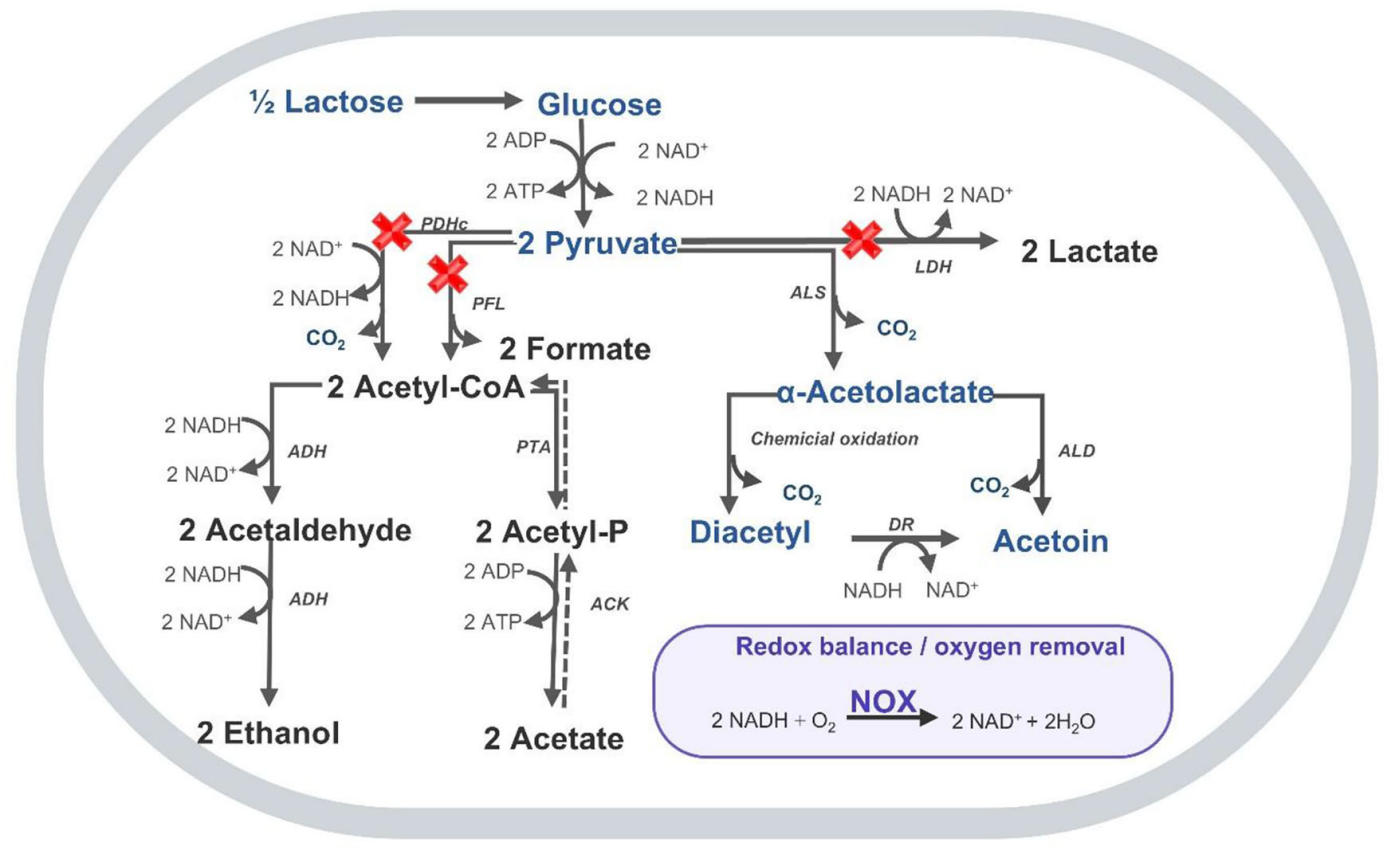
Metabolic pathway for the LDH‐deficient strain *Lc. lactis* RD1M5. ACK, acetate kinase; ADH, alcohol dehydrogenase; ALDB, α‐acetolactate decarboxylase; ALS, α‐acetolactate synthase; LDH, lactate dehydrogenase; NOX, NADH oxidase; PDHC, pyruvate dehydrogenase complex; PFL, pyruvate formate lyase; PTA, phosphoacetyltransferase.

In our laboratory, we have previously developed a strategy for microbial scavenging of oxygen in yogurt, which is based on *Lactococcus lactis* RD1M5, a lactate dehydrogenase (LDH)‐deficient strain obtained through classical chemical mutagenesis (J.‐M. Liu et al. 2020). As shown in Figure 3, there are mainly four pathways for pyruvate metabolism: (1) LDH catalyzes the reversible conversion of pyruvate to lactate, enabling the regeneration of NAD^+^ from NADH and thereby sustaining cellular redox balance. However, *Lc. lactis* RD1M5 is an LDH‐deficient strain, which means this pathway is blocked. Instead, the strain RD1M5 uses the enzyme NADH oxidase to generate NAD^+^ to maintain the redox balance. (2) The accumulated pyruvate is converted into α‐acetolactate by α‐acetolactate synthase (ALS), which is then decarboxylated into acetoin. (3) Normally, pyruvate could also be converted into acetyl‐CoA by the pyruvate dehydrogenase complex (PDHC). However, in milk, PDHC is inactive because it lacks an essential cofactor, lipoic acid. As a result, the conversion of pyruvate into acetyl‐CoA is limited (Zhao and Solem 2024). (4) Pyruvate‐formate‐lyase (PFL) plays a crucial role in the anaerobic metabolism of pyruvate, catalyzing the conversion of pyruvate into acetyl‐CoA and formate. However, both oxygen and low pH block its activity (Hofvendahl et al. 1999; Takahashi et al. 1982). Therefore, three out of four pathways have been blocked. Due to both metabolic limitations of RD1M5 and constraints by the growth environment, once oxygen is depleted, the lack of a functional LDH pathway prevents RD1M5 from regenerating NAD^+^ via lactate formation, leading to a rapid redox imbalance that slows glycolysis and restricts growth. Including RD1M5 in yogurt offers a protective advantage: oxygen is rapidly consumed, after which the anaerobic yogurt environment suppresses RD1M5 growth, thereby minimizing the impact of its metabolic by‐products on yogurt quality while preventing unwanted fungal growth. Preliminary observations suggest that RD1M5 may reduce oxygen levels in milk, highlighting its potential as a bioprotective culture in yogurt systems (J.‐M. Liu et al. 2020). Therefore, the addition of such cultures to yogurt, followed by sealed storage, may contribute to oxygen depletion and potentially extend the shelf life of the product.

Importantly, the concept explored in this study is not isolated from industrial practice. Similar oxygen‐scavenging strategies have already been applied in food systems. For instance, commercial cultures of LDH‐deficient *Lc. lactis* in Bactoferm Rubis (Novonesis) are applied to spray onto the sliced ham surface, followed by packaging, typically at inoculation levels of around 10^7^ CFU/g (Møller and Zuliani 2025). This process results in rapid oxygen depletion and regeneration of NAD^+^, thereby reducing oxidative reactions such as photooxidation and improving color stability in meat products. These findings highlight a shared underlying principle: The modulation of the redox environment through microbial oxygen consumption. Extending this concept to fermented dairy systems, to reduce oxygen availability, may similarly limit the growth of aerobic spoilage microorganisms and enhance yogurt stability, supporting the relevance of our approach.

Besides preventing unwanted fungal growth, depleting oxygen can help promote the growth of starter culture microorganisms used to produce yogurt and cheese, which are known to be hampered by dissolved oxygen in the milk (Ding et al. 2022). Since probiotics, which are frequently added to yogurt products, are also sensitive to oxygen, this oxygen depletion strategy may also offer a novel way to preserve their viability (Miller et al. 2003; Norouzbeigi et al. 2021).

## 4 Strategies for Controlling Yogurt Post‐Acidification

Post‐acidification refers to the continued decline in yogurt pH during storage (from pH 4.0–4.4 decreased to below pH 4.0), driven by the residual metabolic activity of starter cultures, primarily *L. bulgaricus* (Deshwal et al. 2021). This unwanted acid development shortens shelf life and leads to multiple quality defects, including excessive acidity, syneresis, off‐flavors, and reduced viable LAB counts. It also compromises the stability of added probiotics, as the progressively lower pH can reduce cell viability by disrupting membrane integrity, inhibiting metabolic activity (Donkor et al. 2006; Shah 2000; Shah et al. 1995). At the structural level, persistent lactic acid production enhances hydrophobic and electrostatic interactions among milk proteins, promoting casein aggregation, dissolution of colloidal calcium phosphate, and partial rearrangement of the protein matrix. The resulting denser and more rigid gel increases viscosity, firmness, and susceptibility to whey separation (Arab et al. 2023).

To address the challenges associated with post‐acidification, research has converged on four alternative strategies: process optimization, the addition of preservatives, the addition of additives, and the optimization of the yogurt starter culture.

### 4.1 Process Interventions

With the significant development of food processing technologies, several strategies have emerged to prevent post‐acidification in yogurt. These approaches include high hydrostatic pressure, pulsed electric fields, carbon dioxide (CO_2_) treatment, and thermosonication, among others (Deshwal et al. 2021).

High hydrostatic pressure, widely applied in fermented yogurt processing with 200 and 300 Mpa at 40°C, has been reported to suppress post‐acidification by inhibiting key starter enzymes, including LDH, β‐D‐galactosidase, and F_0_F_1_‐ATPase (Guan et al. 2025; Serra et al. 2009). Nevertheless, high hydrostatic pressure treatment also impacts starter culture viability, with *L*. *bulgaricus* generally exhibiting higher pressure sensitivity than *S. thermophilus*. This treatment may reduce viable cell counts below the minimum levels required by regulatory standards in many countries, where yogurt products are typically expected to contain approximately 10^6^–10^8^ CFU/g of live microorganisms (De Ancos et al. 2000; FDA 2023; Kilara 2017).

Pulsed electric field technology employs short, high‐intensity electric pulses to inactivate microorganisms in continuous liquid systems. Typical electric field strengths range from 15 to 50 kV cm^−^^1^, with treatment durations limited to microseconds to milliseconds (Ravishankar et al. 2008). The antimicrobial effect of the pulsed electric field is primarily transient and arises from the exposure of microbial cells to intense electric fields, which induce electroporation of the cell membrane. This process increases membrane permeability and, under sufficiently strong conditions, leads to irreversible membrane disruption, mechanical rupture, and leakage of intracellular contents (Visockis et al. 2025). Using a starter culture composed of *L*. *bulgaricus* and *S. thermophilus*, pre‐treatment with pulsed electric fields under defined conditions (12 kV, 50 pulses, 11 Hz, pulse width 7 µs) has been shown to significantly modify fermentation performance (e.g., by accelerating fermentation, enhancing gel stiffness, and reducing syneresis in the final yogurt). These observations indirectly suggest that pulsed electric field treatment of starter cultures may modulate post‐fermentation acidification in yogurt (Stühmeier‐Niehe et al. 2023).

CO_2_ treatment has been shown to effectively retard post‐acidification in yogurt beverages during cold storage. When chilled, sucrose‐sweetened plain yogurt beverages were carbonated at 0.5 kg CO_2_/cm^2^ and stored at 4.4°C, acid development was significantly slowed, allowing excellent product quality to be maintained for up to 4 months. Under these conditions, a carbonated yogurt beverage with an initial pH of 4.0 exhibited only a modest decrease in pH, reaching 3.85 after 35 days at 4.4°C and 3.72 after storage at 10°C. In contrast, sweetened, plain non‐carbonated control beverages with an initial pH of 4.1 showed substantially greater pH reductions, declining to 3.60 and 3.55 after 35 days at 4.4°C and 10°C, respectively, and became unacceptable within 30 days of storage (Choi and Kosikowski 1985). However, CO_2_‐treated yogurt beverages are not commonly available in commercial markets. To date, one of the few well‐known examples of carbonated yogurt beverages is Doogh, a traditional fermented dairy drink widely consumed across the Middle East from Lebanon to Afghanistan (Koçak and Avşar 2010).

Thermosonication has also been reported as an effective approach to reduce post‐acidification in yogurt‐based products. Studies on Turkish Aryan yogurt demonstrated that thermosonication treatment at 70°C for 5 min mitigated acid development during cold storage. In untreated samples, titratable acidity increased over time and was accompanied by a progressive decline in pH, reaching 4.10 ± 0.20 after 30 days of storage. In contrast, thermosonication‐treated samples maintained stable acidity throughout the storage period, with pH values remaining at 4.22 ± 0.20 after 1 month. This stabilization of acidity is closely associated with reduced microbial load and activity, as thermosonication can cause cell membrane disruption and sublethal injury through the combined effects of heat and ultrasound‐induced cavitation, thereby suppressing continued lactic acid production (Erkaya et al. 2015; Tahi et al. 2021).

However, these approaches require additional application of often rather specialized equipment for mitigating yogurt post‐acidification, which might not be appropriate for small‐ and medium‐sized dairy companies. Introduction of such technologies demands big investments in the purchase/upgrade of production equipment, operator training, and further maintenance costs. For smaller dairies, these financial and operating requirements may make equipment‐based solutions difficult to justify when compared to typical process‐ or culture‐related alternatives.

### 4.2 Preservatives

The use of preservatives is a common strategy to extend the shelf life of yogurt by limiting culture growth, thereby reducing post‐acidification. Recent work shows that PLA is a promising growth inhibitor at low pH. PLA suppresses *L. bulgaricus* by impairing membrane integrity and fluidity, dissipating proton motive potential, and altering cellular ultrastructure. It also inhibits key metabolic enzymes, including the F_0_F_1_‐ATPase, LDH, β‐galactosidase, and 6‐phosphofructokinase, thereby reducing lactic acid formation and weakening acid resistance. Using 1.25 mg/mL of PLA decreased titratable acidity by 5 °T (around 0.045 g lactic acid per 100 mL) after 21 days at 15°C during yogurt storage, thereby reportedly alleviating post‐acidification. However, no corresponding pH values were reported in this study (Zhou et al. 2025). Also, it has been reported that post‐fermentation acidification can be prevented by adding reuterin (9 mM), which suppresses the growth of LAB during refrigerated storage. However, when added in larger amounts, reuterin can disrupt the milk gel (Sun et al. 2025). Other options for preventing post‐acidification include adding Nisin (Sarkar 2016), vanillin (Penney et al. 2004), and microencapsulated crude bacteriocin (Oh et al. 2006).

The prospects for using chemical preservatives to prevent post‐acidification in yogurt remain constrained. First, regulatory limitations restrict the use of some potentially interesting candidates; reuterin, for instance, has not yet been approved for fermented dairy products (Dalal et al. 2023). Second, preservatives are not specific, and their incorporation inhibits not only the target post‐acidification bacteria but also the good fermentation microflora necessary for proper yogurt fermentation (Davidson et al. 2012). In addition, concerns have been raised that certain chemical preservatives may inhibit the growth and alter the composition of the gut microbiota. It has been reported that potassium sorbate (25 mg/mL), sodium bisulfite (0.7 mg/mL), and sodium benzoate (5 mg/L) exhibit strong antimicrobial activity against several gut‐associated bacteria, including *Bifidobacterium longum*, *Enterococcus faecium*, *L. acidophilus*, and *Lc. lactis* subsp. *lactis*. In addition, exposure to sorbate reduced short‐chain fatty acid production in *L. lactis*, *L. acidophilus*, and *B. longum*, compared with untreated controls (de Souza Lopes et al. 2023). Thus, future efforts should target the development of more selective and fermentation‐compatible preservation strategies with reduced adverse impacts on yogurt starter cultures and the gut‐associated microbiota. In the long run, biologically based preservation systems and precision microbial control strategies may offer more specific intervention options for clean‐label yogurt than traditional chemical preservatives do, from a fundamental perspective.

### 4.3 Additives

**FIGURE 4:**
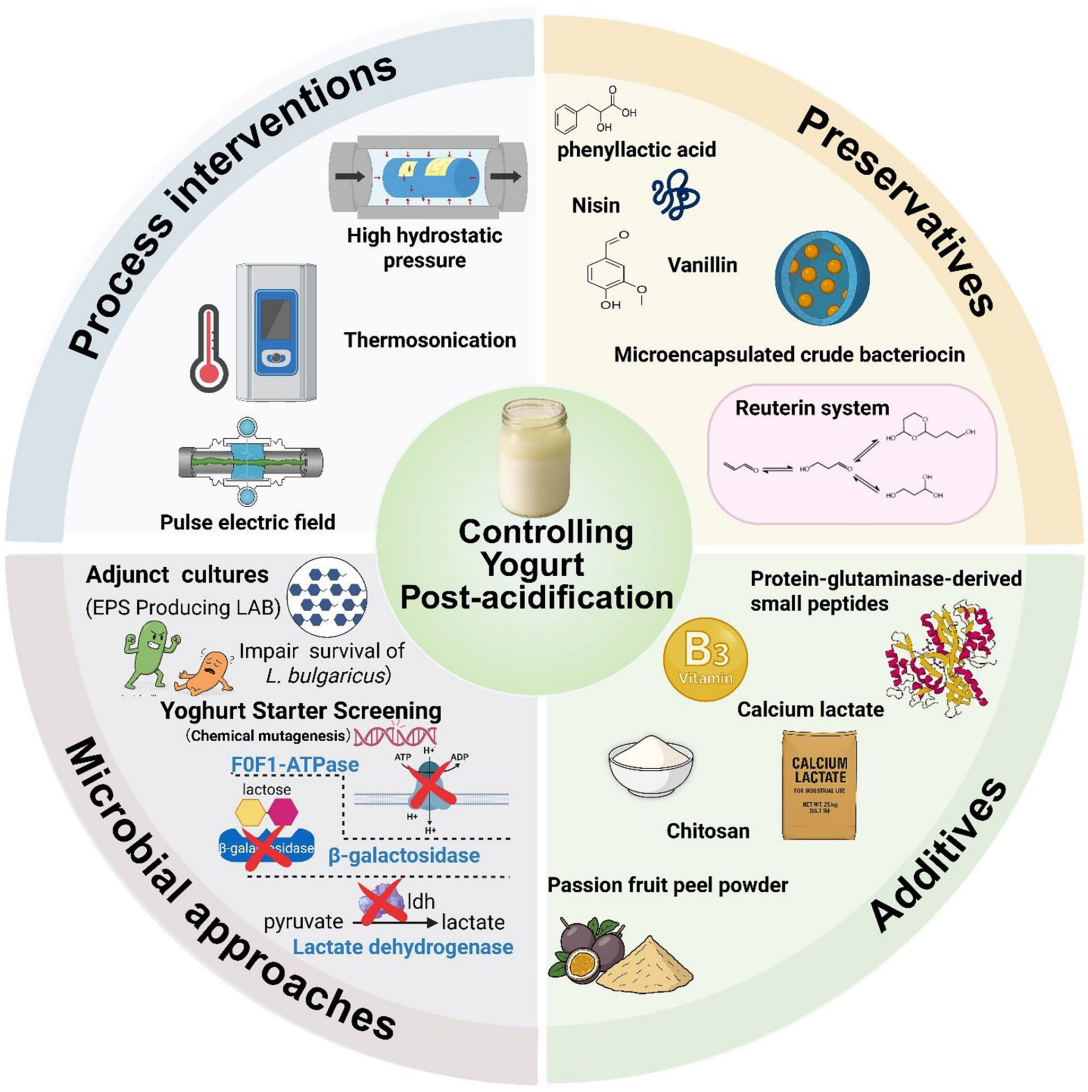
Examples of emerging strategies for controlling yogurt post acidification.

Various ingredients are added during yogurt production to adjust flavor, functionality, shelf life, and texture. As shown in Figure 4, we have listed potential additives that can delay yogurt post‐acidification. It has been reported that adding calcium lactate can provide buffering capacity during milk fermentation, to stabilize yogurt pH, and delay post‐acidification without adversely affecting starter culture activity, fermentation time, or sensory quality (M. Yang et al. 2011). As a lactose‐derived component, it moderates acid accumulation while maintaining starter balance and overall product stability. It has been reported that treatment of set‐type yogurt with 0.06 U/mL of protein‐glutaminase–derived small peptides significantly reduces post‐acidification by limiting lactic acid accumulation while simultaneously maintaining bacterial viability and enhancing the product's physical properties (J. Yang et al. 2025). Recent studies show that incorporating chitosan, particularly in nanoparticle form, can effectively reduce post‐acidification in yogurt. Chitosan‐fortified samples maintained higher pH values during cold storage, suggesting reduced lactic acid formation. Notably, lower chitosan nanoparticle concentrations (2.5 mg in 100 mL milk) provided these benefits without compromising sensory quality, highlighting chitosan as a promising adjunct for controlling post‐acidification (Salama and Shahein 2023). It has also been reported that incorporating passion fruit peel powder throughout yogurt fermentation helps mitigate post‐acidification in full‐fat products. After 14 days of storage, all full‐fat yogurts supplemented with the powder exhibited higher pH values than their respective controls, indicating reduced acid development. However, this effect was not observed in low‐fat yogurt, suggesting that its efficacy may depend on the yogurt's fat content (Santo et al. 2012). The authors proposed that, in full‐fat yogurt, fatty acid consumption may partially contribute to alkalinization and counteract yogurt acidification, whereas this is less pronounced in low‐fat systems. In contrast, degradation of pectin from passion fruit peel into uronic acids may further promote acidification in low‐fat yogurt, thereby offsetting the pH‐stabilizing effect.

### 4.4 Microbial Approaches

Among the microbial strategies proposed to mitigate post‐acidification in yogurt, we believe the three most promising approaches are the use of adjunct cultures and the non‐GMO (genetically modified organism) modification of *L. bulgaricus*.

#### 4.4.1 Reduce the Proportion of Acidification‐Related Bacteria in Yogurt

Recently, advances in yogurt production have introduced innovative strategies to overcome key challenges associated with post‐acidification and microbial spoilage during storage. Traditional yogurt production relies on thermophilic starters, mainly *S. thermophilus* and *L. bulgaricus*, which, although effective in fermentation, often lead to excessive acidification and shortened shelf life during storage. A novel approach has been proposed that employs non‐growing LAB at elevated temperatures (∼51°C), enabling acidification without promoting further bacterial growth (Gu et al. 2025). By increasing the inoculum size, it is possible to achieve the target pH (4.6) efficiently, even at high temperatures where bacterial proliferation is inhibited. This method reduces the risk of post‐acidification, maintaining the yogurt pH above 4.0 after 40 h incubation at 42°C using starter culture YC380 to simulate post‐acidification conditions. It also extends shelf life and minimizes spoilage caused by yeasts and molds. Moreover, an economic advantage of the method is that five‐fold less yogurt culture is needed, which contributes to lowering the overall cost of producing the yogurt. The reduction in production cost is mainly related to reduced dependency on DVS starter cultures, which are commonly used for large‐scale yogurt processing but are more expensive. For instance, one 50 U bag of DVS yogurt starter culture is enough to produce direct 250 L of yogurt. On the other hand, the referred method first utilizes DVS to create a bulk starter culture and that is then added into yogurt milk at about 10%–20%. This strategy allows the same amount of DVS culture to generate approximately 1250–2500 L of yogurt in total, leading to a significant reduction in starter consumption and production costs. In many modern dairy facilities, the additional processing costs associated with bulk starter preparation, compared to classical processes are regarded as minimal because of the need for extensive heat treatment of yogurt milk, facilitating industrial heat‐exchanger systems, which limit thermal energy losses. Overall, this novel method represents a promising development for producing high‐quality, cost‐effective, and longer‐lasting yogurt, benefiting both the dairy industry and consumers.

A similar strategy has been reported previously for milk cultured with *L. rhamnosus*, where mild heating (46°C for 1 min) reduced acid formation by 70% by impairing growth and metabolic activity, while preserving a high viable count of *L. rhamnosus* (C. Zhang et al. 2019). It has also been shown that freezing followed by mild heating can selectively attenuate the acid‐producing activity of *L. paracasei*, *L. bulgaricus*, and *S. thermophilus*, while preserving desirable flavor development (Zhu et al. 2017). Overall, these methods represent promising developments for producing high‐quality, cost‐effective, and longer‐lasting yogurt, benefiting both the dairy industry and consumers.

#### 4.4.2 Adjunct Cultures for Mitigating Post‐Acidification

One strategy is to inhibit *L. bulgaricus* growth, thereby reducing post‐acidification and accompanying syneresis. It was found that *Lactobacillus plantarum* WCFS1, precultured under sublethal conditions (under conditions of 1.5% NaCl at pH 4.5 and 4.5% NaCl combined with either pH 4.5 or 6.5) and added to yogurt, could suppress excessive growth of *L. bulgaricus* and thereby reduce post‐acidification; the stress‐adapted cells apparently alter competitive interactions and shift metabolite formation, leading to impaired survival of *L. bulgaricus* (Settachaimongkon et al. 2016). Also, *Bifidobacterium animalis* subsp. *lactis* BL O4 displays low acid‐producing activity at refrigeration temperatures, with yogurt fermented in combination with *S. thermophilus* exhibiting comparatively low levels of post‐acidification during storage (Damin et al. 2008).

#### 4.4.3 Non‐GMO Approaches for Reducing Post‐Acidification of *L. bulgaricus*

Reducing the acid production ability of *L. bulgaricus* is an efficient way to avoid post‐acidification of yogurt. Here, we describe how classical chemical mutagenesis can be harnessed for this purpose.

The F_0_F_1_‐ATPase, also referred to as the H^+^‐ATPase, plays a central role in maintaining intracellular pH homeostasis by actively extruding protons (H^+^) from the cell. This mechanism allows *L. bulgaricus* to remain metabolically active under acidic conditions, thereby contributing to post‐acidification in yogurt (Bonza et al. 2016; Yue et al. 2022). Several studies have shown that mutants with reduced F_0_F_1_ ATPase activity are less acid‐tolerant and stop fermenting earlier, which helps prevent post‐acidification. Such mutants can be obtained by taking advantage of an observed negative correlation between F_0_F_1_‐ATPase activity and bacterial resistance to neomycin (Yamamoto et al. 1996). In this way, spontaneous acid‐sensitive mutants of *L. bulgaricus* have been isolated, which display reduced post‐acidification capacity (Ongol et al. 2007; X. Wang et al. 2013). Another approach for obtaining mutants with defective F_0_F_1_‐ATPase is to carry out adaptive evolution in the presence of N, N‐dicyclohexylcarbodiimide, which is a specific inhibitor of the F_0_F_1_‐ATPase (X. Wang et al. 2013).

In *L. bulgaricus*, lactose is metabolized via homolactic fermentation. Lactose is first transported into the cell by a permease, after which β‐galactosidase hydrolyzes it into 1 molecule of glucose and 1 molecule of galactose (W. Zhang et al. 2012). Strains lacking β‐galactosidase activity have been identified. Since such strains are unable to metabolize lactose, they are also unable to cause post‐acidification in yogurt (Mollet and Delley 1990). It is also reported that mutations in the β‐galactosidase gene can confer strong enzymatic activity at 40°C; however, during low‐temperature storage, the activity weakens or is even lost (Brignon et al. 2005). To obtain β‐galactosidase mutants, chemical mutagenesis with hydroxylamine or methoxylamine was applied to the β‐galactosidase gene carried on an *Escherichia coli* expression vector. Mutants showing sensitivity to cold, heat, low pH, low magnesium concentrations, or the presence of urea were then isolated by screening for reduced color development on β‐galactosidase indicator plates (Yoast et al. 1994). This method can also serve as a model for generating *L. bulgaricus* β‐galactosidase mutants. To screen for absence of β‐galactosidase activity, the chromogenic compound X‐gal can be used, which gives rise to a blue colored compound following hydrolysis by the β‐galactosidase enzyme(Mollet and Delley 1990; Tadesse et al. 2022).

Another target for reducing post‐acidification is lactate dehydrogenase (LDH). Pyruvate, the end product of glycolysis, is reduced to lactic acid by LDH. There are multiple LDHs in LAB, and *L. bulgaricus* possesses both D‐LDH and L‐LDH, with D‐LDH being the predominant isoenzyme. As a result, more than 90% of pyruvate is directed toward D‐lactate production (Razeto et al. 2002). LDH plays a pivotal role in regulating the growth and acidification capacity of LAB. In principle, reducing or inhibiting LDH activity in *L. bulgaricus* would be expected to suppress lactic acid production; however, to date, no experimental studies have reported the use of a non‐GMO approach for this purpose. Recently, a CRISPR/Cas9‐mediated disruption of the *L. bulgaricus* D‐LDH gene was reported. Yogurt produced with the genetically modified strain displayed markedly reduced acidification, with final pH values ranging from 5.1 to 5.8, in contrast to the typical pH of 4.0–4.4 observed in conventional yogurt. These findings highlight the crucial role of LDH in *L. bulgaricus* in driving lactic acid production and contributing to yogurt's characteristic acidity (Hasan et al. 2024). To obtain LDH‐deficient *L. bulgaricus* strains using non‐GMO approaches, classical chemical mutagenesis can be employed. Following mutagenesis, LDH‐deficient colonies may be screened on MRS agar supplemented with 0.5% calcium carbonate, where reduced acid production results in smaller or less distinct dissolution zones (Hasan et al. 2024). Additional differential media can also aid in identifying low‐acid‐producing mutants, such as MRS‐TTC (2,3,5‐triphenyl tetrazolium) agar, on which acid‐deficient colonies appear red (J.‐M. Liu et al. 2020; Monnet et al. 2000), and PCA‐BCP (plate count agar with bromocresol purple) agar, where decreased acidification produces colonies that are less yellow and more purple (Lee and Lee 2008). It is also reported that low‐post‐acidifying *L. bulgaricus* mutants can be obtained through diethyl sulfate mutagenesis, followed by selection for growth in milk but not in low‐pH MRS media. These mutants exhibit reduced acidification resistance, stable at 11.9%, compared to the original strain, likely due to impaired acid tolerance and reduced metabolic activity under acidic conditions (S. Zhang et al. 2012).

To sum up, industrial robustness, scalability, and strain in situ compatibility of these microbial approaches under real yogurt production and storage conditions should be the focus of future research. Specific consideration should be given to achieving a balance between post‐acidification ability, fermentation efficiency, strain stability, and product quality. In the long term, advances in microbial ecology, adaptive evolution, and systems biology are expected to drive the development of next‐generation yogurt strains for production of clean‐label and minimally‐processed yogurt. On another note, future dairy production systems may benefit from this work, as it indicates that AI‐assisted fermentation monitoring, combined with predictive metabolic modeling, can help design approaches to optimize both fermentation and storage stability in industrial yogurt.

## 5 Lactase Treatment for Lactose Reduction and Natural Sweetness Enhancement in Yogurt

Almost 70% of the world's population has a poor ability to metabolize lactose in milk due to naturally low lactase activity in their gut (Swagerty et al. 2002). Fermented dairy products such as yogurt are well tolerated by lactase‐deficient subjects, a phenomenon that is attributed to the β‐galactosidase activity associated with live yogurt cultures (Savaiano et al. 1984). Regulatory definitions of lactose‐free products remain heterogeneous across regions. While no universal standard exists, several countries define “low‐lactose” products as containing less than 1 g lactose per 100 g, and “lactose‐free” products as containing below 10–100 mg lactose per 100 g (Mangan et al. 2019). Nevertheless, lactose‐free and lactose‐reduced yogurts have gained substantial market traction and are often sold at a premium, targeting consumers perceived to have lactose intolerance (Dekker et al. 2019). In parallel, many commercial yogurt products, particularly flavored and drinking yogurt, contain substantial amounts of added sucrose to improve sweetness and consumer acceptability since lactose exhibits substantially lower sweetness than sucrose. However, increasing awareness of the adverse health effects of excessive sugar consumption including obesity and type 2 diabetes has prompted international initiatives by organizations such as the Food and Agriculture Organization (FAO) of the United Nations and the WHO to reduce dietary sugar intake (Herman et al. 2015). Within this context, strategies that enhance intrinsic sweetness without added sugars and even reduce lactose in yogurt are of growing interest.

### 5.1 Removing Lactose From Milk‐Membrane Filtration Processes

Membrane filtration technologies, particularly microfiltration and ultrafiltration (UF), are widely applied in the dairy industry to selectively adjust lactose levels in milk and dairy products. In industrial dairy processing, during 3 × UF (volume is reduced by a factor of 3 after UF), one of the most commonly applied concentration factors, where approximately 66%–67% of lactose is transferred into the permeate, while the remaining 33%–34% is retained in the UF milk retentate (McSweeney and Fox 2009). A non‐enzymatic alternative for low‐lactose milk powder preparation is a combination of UF, electrodialysis, and a nanofiltration‐based membrane processing system. This method yielded milk powder with < 0.2% lactose under optimized operating conditions, while allowing the recovery of ∼95.7% lactose (H. Zhang et al. 2020). Membrane filtration performance is governed by permeate flux, membrane selectivity, and fouling resistance. However, concentration polarization and membrane fouling are the main limitations for large‐scale UF processing, with solutes accumulating at or within the membrane pores, decreasing separation efficiency over time (Don et al. 2025). Also, UF‐induced changes in milk composition including increased fat and protein content, reduced lactose content, altered mineral content, and losses in B vitamins and free amino acids may affect the yogurt manufacturing process, e.g. the growth of the LAB is affected when the concentration factor is above 5 (Premaratne and Cousin 1991).

An alternative route to reducing lactose can be achieved by integrating membrane filtration with complementary processing technologies. For instance, Valio Ltd. (Helsinki, Finland) has developed an integrated processing strategy with a combination of chromatography, membrane separation, and enzymatic hydrolysis in the industrial manufacture of lactose‐free milk (A. Li et al. 2023). However, with such equipment, costs are usually higher; reported annual operating costs for industrial UF systems generally range from £1000 to £2000 per cubic meter per day of processing capacity. Energy demand represents the largest operational expense (30%–40%), followed by membrane replacement (25%–35%), chemical cleaning and clean‐in‐place (CIP) procedures (15%–20%), and labor required for maintenance and monitoring (10%–15%; Bokkel 2026).

### 5.2 Commercial Lactase Is Used for Lactose‐free or Reduced Yogurt

Yogurt contains 3.5%‐4% lactose, with a low sweetness level, only 16% that of sucrose. By hydrolyzing lactose, the sweeter sugars glucose and galactose are released. In terms of sweetness, it is desirable to hydrolyze only part of the lactose; hydrolyzing 70% of the lactose increases sweetness by an amount corresponding to 2% sucrose, and this degree of hydrolysis provides the maximum sweetness (Harju et al. 2012). Another benefit of lactase hydrolysis in milk and dairy products is the formation of galacto‐oligosaccharides (GOS). GOS are mainly produced by enzymatic transgalactosylation catalyzed by β‐galactosidase, using lactose as the substrate. These compounds selectively promote the growth of beneficial gut microbiota, thereby contributing to improved intestinal health (Costa et al. 2025).

**FIGURE 5:**
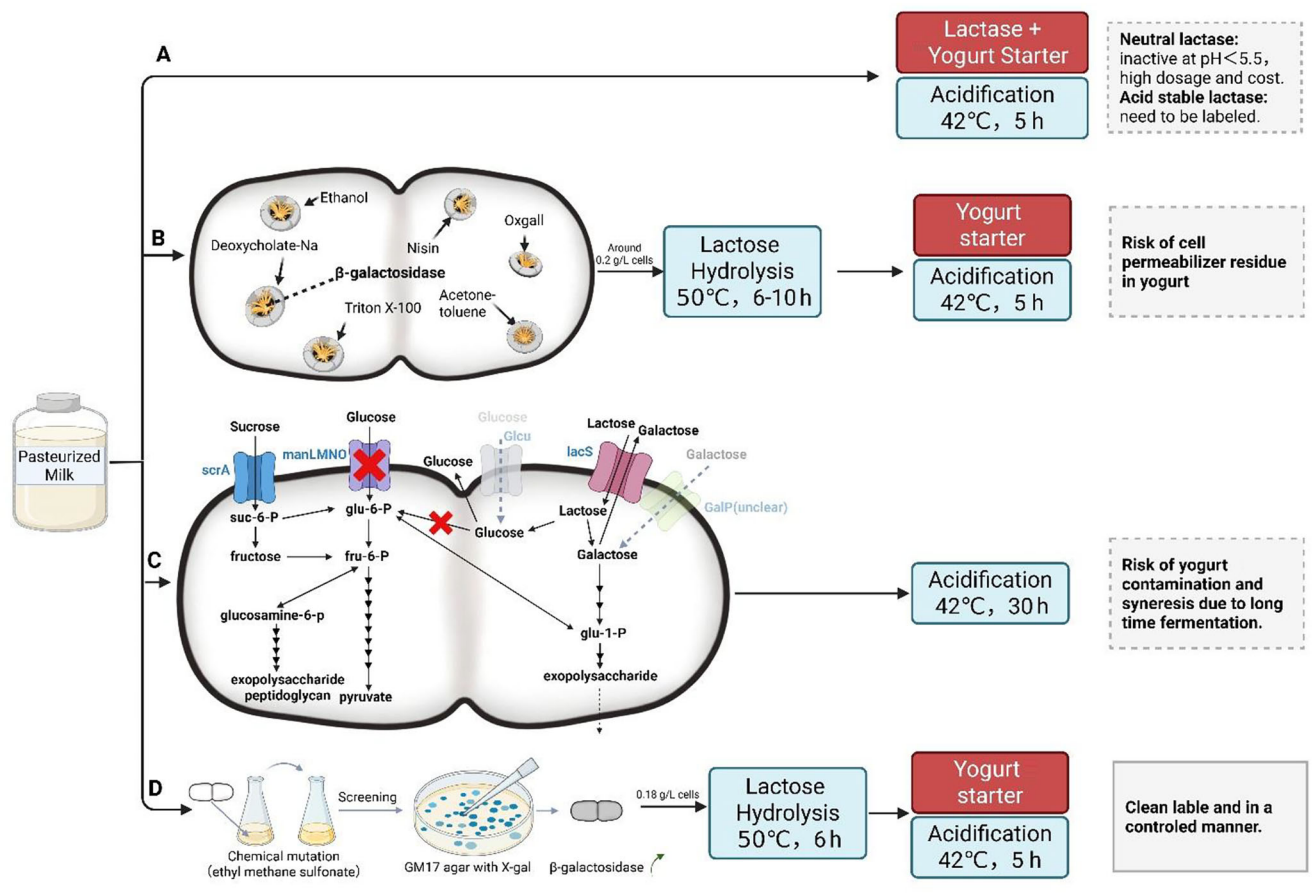
Different methods for making lactose‐free yogurt with enhanced sweetness through lactose hydrolysis. (A) Commercially available process for producing lactose‐free yogurt by the addition of lactase in combination with yogurt starter cultures. (B) Proposed workflow for lactose‐free yogurt production employing different chemically permeabilized whole‐cell lactase catalysts. (C) Proposed workflow for lactose‐free yogurt production employing *S. thermophilus* mutations that affect glucose phosphorylation and the phosphotransferase system for glucose. The genes indicated in the figure are: scrA, sucrose phosphotransferase system component II; manLMNO, glucose/mannose PTS IIABCD; lacS, lactose permease; glcU, glucose permease (predicted); and galP, putative galactose permease (unclear). glu‐1‐P, glucose‐1‐phosphate; glu‐6‐P, glucose‐6‐phosphate; fru‐6‐P, fructose‐6‐phosphate; suc‐6‐P, sucrose‐6‐phosphate (adapted from (Sørensen et al. 2016). (D). Proposed workflow for lactose‐free yogurt production employing *S. thermophilus* whole‐cell catalysts with enhanced lactase activity.

As shown in Table 2, many lactase (β‐galactosidase) variants are commercially available and can be divided into neutral and acid lactases (Dekker et al. 2019). The addition of β‐galactosidase treatment increases the production cost of fluid milk by approximately $0.06–0.08 per liter (McSweeney and Fox 2009). As shown in Figure 5A, the enzyme used for the production of lactose‐free dairy products has traditionally been the neutral β‐galactosidase derived from the dairy yeast *Kluyveromyces lactis* or *K. marxianus*, and also *Bifidobacterium bifidum*, which is inactivated by the low pH in yogurt and thus is added alongside the culture (Agarwal et al. 2022). However, the natural lactase is not particularly efficient, and it has been reported that 80% hydrolysis of lactose in milk can be accomplished in 48 h at 4°C when using 25 mg/L of enzyme, and to achieve 95% hydrolysis, 100 mg/L of lactase was needed (Dorau et al. 2021). In contrast, lactases derived from fungal sources such as *Aspergillus oryzae* typically exhibit a lower pH optimum (“acid lactases” work around pH 2.5–5.4) and can therefore be added directly during yogurt fermentation (Saqib et al. 2017). However, the use of such enzymes generally requires the declaration of lactase in the final product, which is disadvantageous for clean‐label yogurt formulations that are commonly preferred in the dairy industry. Notably, several commercially available lactases are produced using genetically modified microorganisms, For instance, a lactase originating from *B.ifidobacterium bifidum* and recombinantly expressed in *Bacillus licheniformis* has been commercialized under the trade names Saphera and NOLA Fit (Novonesis, Denmark; Dorau et al. 2021). While such recombinantly produced enzymes do not require labeling as GMOs in the final product under European regulations, they are generally not accepted in GM‐free product concepts. Consequently, the use of these enzymes remains limited among manufacturers targeting GM‐free or Verband Lebensmittel Ohne Gentechnik (VLOG) compliant dairy products, regardless of their technological performance (VLOG 2026).

**TABLE 2:** Representative commercial lactase or lactase function yogurt starter used in dairy products.

Product name	Enzyme source	Function	Optimized conditions for application	Manufacturer	Reference
Maxilact Next	*Kluyveromyces lactis*	Intended for use in hydrolyzed milk and milk products, the final enzyme preparation is non‐GMO, despite the use of genetically modified microorganisms in its production.	At a pH value of > 5.5	DSM Food Specialties	Dsm‐firmenich (2026b)
GODO YNL2 LACTASE	*Klyveromyces lactis*	Reduced‐lactose cultured milk products such as yogurt, yogurt drinks, and cheese products	Its optimal pH is in the neutral range	DuPont's Nutrition & Biosciences Business	Dairy Connection (2026a)
Lactozym pure 6500	*Kluyveromyces lactis*	Pre‐processing of milk or yogurt	Neutral lactase has an optimal pH between 6 and 8 and an optimal temperature of approximately 37°C.	Novonesis	Novonesis (2026a)
Lacta‐free enzyme	*Aspergillus oryzae*	Specifically formulated for lactose‐free milk and other dairy products within the “Vivi‐Tek” product range.	For milk, the hydrolysis process is carried out at 40 ± 2°C for 5.0 ± 0.5 h.	Biochem srl.	Biochem 2026; Titov et al. (2020)
Lactase	*Aspergillus oryzae*	Rapid lactose hydrolysis under acidic conditions eliminates lactose in milk, yogurt, and whey‐based drinks	Active at pH 4.0–6.5 (optimum ∼5.0) Optimal temperature: 35°C–55°C	Chibio Biotech	Chibo (2026)
Food enzyme β‐ galactosidase	*Papiliotrema terrestris*	Food enzyme β‐galactosidase from a non‐genetically modified strain of *Papiliotrema terrestris*.	The enzyme has a temperature optimum around 70°C (pH 6.0) and a pH optimum around pH 5.0 (40°C).	Amano Enzyme Inc	Lambré et al. (2024)
Saphera	The gene for lactase originates from *Bifidobacterium bifidum*, but the enzyme is produced through fermentation using a genetically modified *Bacillus licheniformis*.	Specifically for the dairy industry, including milk, yogurt, and infant formula.	Optimal operating conditions of pH 5.7–6.4 and temperatures between 40°C and 50°C	Novonesis	Univar Solutions (2026)
NOLA Fit550	*Bifidobacterium bifidum*	It is produced by submerged fermentation on a vegetable‐based substrate using a selected strain of *Bacillus licheniformis*, which is maintained under contained conditions and is not present in the final product. Suitable for fermented milk and cheese manufacture without the off‐flavors typical of lactose‐free UHT/ESL milk products.	Optimized for pH 5.4–7.0 and temperatures between 35°C and 50°C	Novonesis	Czyzewska and Trusek (2021)
Sweety	Yogurt starter culture with selected strains of *S. thermophilus* and *L. bulgaricus*	Employs a natural starter culture solution, meeting requirements for organic, non‐artificial, and VLOG labeling. Maintaining sweetness while reducing added sugar by 0.5–1 g per 100 g of yogurt	Yogurt fermentation conditions	Novonesis	Novonesis (2026b)

### 5.3 Emerging and Innovative Methods for Producing Lactose‐Free/Reduced Yogurt

As early as 1973, Wierzbicki and Kosikowski demonstrated the great potential of various microorganisms grown in whey to hydrolyze lactose, and they found that freeze‐dried cells (0.8 g/L) of *L. helveticus* could hydrolyze 80% of lactose in whey in 5 h under optimal conditions (Wierzbicki and Kosikowski 1973). As shown in Figure 5B, there have been many attempts to use permeabilized or disrupted cells of various LAB for hydrolyzing lactose such as using nisin, ethanol, deoxycholate‐Na, oxgall, triton X‐100, and acetone–toluene (Q. Wang et al. 2020). Although these have not yet achieved commercial success, their potential is obvious. In general, to achieve optimal lactase activity, cells need to be permeabilized, as lactase is located inside the cells.

To address this challenge, Tadesse et al. (2022) developed a solution based on a natural *S. thermophilus* strain able to hydrolyze lactose concurrently with growth, a simple dairy‐compatible solution with great potential for optimization. As shown in Figure 5C, to enhance the lactose‐hydrolyzing activity of *S. thermophilus*, chemical mutagenesis was applied, followed by screening for mutants with increased β‐galactosidase activity. The selected mutant exhibited a 2.4‐fold increase in β‐galactosidase activity, compared with the parent strain. Notably, this strain hydrolyzed lactose as a whole‐cell biocatalyst without any cell permeabilization treatment. At 50°C, only 0.18 g/L of ST30‐8 cells was required to hydrolyze 30 g/L lactose within 6 h (Tadesse et al. 2022). This approach enables the production of low‐lactose sweet yogurt without the use of expensive purified lactase, while fully meeting consumer demand for clean‐label yogurt products.

Another approach for achieving sweetness in yogurt was developed by Sørensen et al. 2016, which is based on mutants of *S. thermophilus* and *L. bulgaricus* with altered lactose metabolism, a solution that has been commercialized. As shown in Figure 5D, this solution relies on strains that secrete glucose rather than galactose due to mutations that affect glucose phosphorylation and the phosphotransferase system (PTS) for glucose and mannose. It was demonstrated that when milk was fermented for a prolonged period, almost 30 h, little lactose remained. Notably, these strains were obtained without the use of recombinant DNA technology, highlighting their industrial applicability for producing low‐lactose, naturally sweeter yogurt (Sørensen et al. 2016). The commercial viability of this solution remains to be seen, however, this culture exhibits weak acidification, requiring approximately 10 h to reach pH 4.6, which is far from 4 to 6 h that a normal yogurt fermentation takes. This also introduces an unnecessary risk of contamination with unwanted microorganisms. As shown in Table 2, Novonesis has also developed a commercial yogurt starter culture (composed of *S. thermophilus* and *L. bulgaricus*), Sweety, which is reported to maintain perceived sweetness while reducing added sugar by 0.5–1 g per 100 g of yogurt. This example further demonstrates the potential and value of optimizing yogurt starter cultures for the production of lactose‐free or reduced yogurt.

For lactose‐free or reduced‐lactose yogurt, we consider reduced‐lactose yogurt to have greater potential for future development. Reducing lactose intake to extremely low levels may compromise potential health benefits, as lactose has been shown to act similarly to soluble fiber in lactose‐deficient individuals. It can be fermented by gut microorganisms, leading to a reduction in colonic pH, and consumption of lactose in dairy products has been suggested to be an important protective factor against the development of colorectal cancer (Thornton 1981). Moreover, while complete lactose hydrolysis is not strictly necessary to accommodate lactase‐deficient consumers, partial lactase treatment can increase sweetness and thereby reduce the need for added sugar in yogurt formulations (Dekker et al. 2019).

Microbial strategies that redirect lactose metabolism offer an efficient approach to enhance the intrinsic sweetness of yogurt and can enable production of yogurt with less added sugar, that fits into the clean label and health‐conscious consumer markets. However, changes in lactose metabolism might affect fermentation kinetics, texture formation, and process robustness, underscoring the need for additional strain and process optimization for industrial applicability. It is worth noting that the development of low‐lactose yogurt strongly depends on a combined approach between starter culture producers and yogurt manufacturers: while the former need to pursue the optimization of metabolic pathways in order to obtain strains of LAB with high β‐galactosidase activity, together with identifying strain combinations favoring assimilation capacities and fermentation of lactose and sucrose; the latter must adapt fermentative procedures and process conditions such as per time/temperature reaction toward lactose hydrolysis targets by accurately controlling contamination risks associated with over‐fermentation or supplementary heat treatments. A unified strain–process co‐development framework will therefore be critically needed to achieve microbiological stability, product quality, and process economics in large‐scale industrial settings.

## 6 Yogurt Bulk Starters: Cost‐Efficient Alternative to DVS Cultures

### 6.1 Protocooperation—Is That Always Essential?

The symbiosis between *S. thermophilus* and *L. bulgaricus* in yogurt fermentations is well documented and is a complex, extensively researched phenomenon (Sieuwerts 2016). In consensus, *S. thermophilus* typically grows first in milk, consuming oxygen and producing formic acid and folic acid, which serve as essential cofactors for purine biosynthesis, which *L. bulgaricus* cannot synthesize (Noorhadi et al. 2025). After oxygen is consumed, *L. bulgaricus* becomes metabolically active and expresses a potent proteolytic activity that efficiently releases peptides that *S. thermophilus* benefits from, as most yogurt *S. thermophilus* strains lack a cell‐envelope‐bound protease (Ulmer et al. 2022). These amino acids support the second growth phase of *S. thermophilus*, which in turn results in the production of sugars and certain branched‐chain amino acids that further benefit *L. bulgaricus*. Through this reciprocal exchange of essential metabolites carbon sources (glucose and galactose produced from *S. thermophilus* hydrolyze lactose), amino acids, vitamins, and improved environmental conditions, the two bacteria fulfill each other's nutritional requirements and drive an efficient milk fermentation (S. Yang et al. 2025).

However, this protocooperation is not always essential, as some *S. thermophilus* strains possess proteolytic capacity, enabling them to grow rapidly in milk in pure culture. Settachaimongkon (2014) reported that only the non‐proteolytic *S. thermophilus* strain (Prt^−^) exhibited true proto‐cooperation with *L. bulgaricus*. By contrast, the protease‐positive *S. thermophilus* strain (Prt^+^) was able to grow independently in milk, reaching a viable count of 1.26 × 10^8^ CFU/g, which is comparable to the population obtained in a mixed culture of *S. thermophilus* Prt^−^ and *L. bulgaricus* (3.16 × 10^8^ CFU/g). These findings highlight that the presence or absence of cell‐wall protease activity significantly alters the metabolic dependence between the two yogurt starters and modulates the extent of their cooperative growth. Additionally, many studies utilize a single strain of *L. bulgaricus* to produce yogurt (Aghababaie et al. 2015; W. Liu et al. 2016; Zhou et al. 2025). Despite this knowledge, the importance of protocooperation is often stressed, even though consumer preference is for less acidic fermented milks with low or no *L. bulgaricus* content. It should also be noted that wild‐type *L. bulgaricus* strains mostly produce D‐lactic acid, which is perceived as more acidic than L‐lactic acid, necessitating the use of more added sugar in sweetened yogurts that most consumers prefer, and furthermore, that the WHO has recommended that the intake of D‐lactic acid should be restricted due to potential toxic effects (Pohanka 2020).

### 6.2 Revisiting Bulk Starter Cultures for Sustainable Yogurt Manufacturing

DVS starter cultures have become the preferred option in modern yogurt manufacture because they provide superior batch‐to‐batch consistency, reduced phage susceptibility, and simplified operations by eliminating daily propagation steps (Admix 2025). Produced under controlled industrial conditions, DVS starters ensure stable strain ratios and reproducible acidification behavior, thereby supporting product standardization across facilities (Novonesis 2025). These characteristics are particularly important for large‐scale industrial yogurt production, where strict quality control, product uniformity, and process reliability are essential. Nevertheless, bulk starters remain a viable approach, particularly in settings where flexibility and cost considerations are important. Their performance can be improved by strategies that modulate the balance between *S. thermophilus* and *L. bulgaricus*, such as elevating incubation temperature to favor *S. thermophilus* (Gu et al. 2025), or by supplementing sucrose (Bills et al. 1972), which promotes *S. thermophilus* growth and reduces the growth of *L. bulgaricus*. Another strategy for maintaining the balance between the two yogurt starter bacteria in bulk starter systems involves controlling dissolved oxygen levels.

If bulk starters are well prepared and the growth kinetics of *S. thermophilus* and *L. bulgaricus* are carefully controlled, this approach could substantially reduce yogurt production costs for the dairy industry. However, the broader industrial implementation of bulk starter systems still faces several practical challenges. Compared with DVS cultures, bulk starters are generally more susceptible to batch‐to‐batch variability, bacteriophage contamination, microbial instability, and inconsistencies in acidification behavior, all of which may negatively affect yogurt quality and process (Admix LTD 2018). However, many of these challenges can be effectively mitigated in industrial practice through appropriate hygienic management, phage‐control strategies, optimized fermentation conditions, and improved process monitoring systems. Consequently, many dairy manufacturers remain hesitant to conduct large‐scale in‐house optimization trials due to concerns regarding contamination risks, product inconsistency, and potential economic losses. Furthermore, commercial starter‐culture suppliers have limited incentive to deviate from the highly profitable DVS culture business model. As a result, industrial research and technological development related to bulk starter systems remain relatively limited despite their potential economic advantages. In the short term, these constraints may hinder the large‐scale use of bulk starter systems in industrial yogurt production with higher degrees of standardization over a longer term. In any case, the strong enforcement of cost‐reduction and sustainability measures in the long run is expected to rekindle interest in optimized bulk starter technologies as a complementary or alternative to traditional DVS systems.

## 7 Regulatory Approval of Ingredients Used in Yogurt Production

The manufacture of yogurt involves a range of ingredients including starter cultures, food additives (stabilizers, sweeteners, colorants, emulsifiers, and preservatives), enzymes, and bulk starter media components, each regulated differently depending on the jurisdiction of sale. For yogurt, the regulatory framework in place is often similar in different countries: Substances to be used in yogurt must be demonstrated to be safe, functionally justified, and will not mislead consumers prior to approval. However, the regulatory mechanisms enacted to satisfy these requirements vary dramatically across regions (Mukherjee et al. 2022). As shown in Table 3, we present an overview of the key regulatory systems affecting yogurt ingredients in the United States, EU, and other jurisdictions, with the achievement of an international baseline developed by the Codex Alimentarius Commission (CAC). This section also provides some legal references for local yogurt manufacturers to conduct export trade.

**TABLE 3:** Comparison of international regulatory frameworks governing yogurt starter cultures, additives, enzymes, and health claims.

Region/country	Regulatory characteristics	Starter culture requirements	Additives and enzymes	Health/nutrition claims	Key regulations	Regulatory strictness
United States (FDA)	Standardized food system with relatively flexible implementation	Yogurt produced with a culture consisting of *S. thermophilus* and *L. bulgaricus*; additional LAB may also be added	Broad range of optional ingredients permitted, including sweeteners, stabilizers, preservatives, and enzymes under GRAS or FDA approval systems	Nutrition and structure/function claims permitted if scientifically substantiated	21 CFR § 131.200 21 CFR Part 170 21 CFR Part 184	Moderate to low
European Union (EFSA/EC)	Strict “positive list” and premarket authorization system	Commonly follows Codex definitions; QPS system used for microbial safety evaluation	Only explicitly authorized additives and enzymes may be used; preservatives in plain fermented are highly restricted	Very strict scientific substantiation required for all health claims	No 1333/2008 No 1332/2008	High
Codex Alimentarius	International reference standard for harmonization and trade	Defines yogurt using *S. thermophilus* and *L. bulgaricus* as characteristic cultures	Provides internationally accepted framework for permitted additives	Offers general guidance for nutrition and health claims rather than legally binding rules	CXS 243–2003	Moderate
Australia/New Zealand (FSANZ)	More flexible risk‐based framework.	Similar to Codex requirements	Positive‐list system for additives and enzymes	Extensive regulation of GI, lactose‐free, protein, and nutrient‐content claims	FSANZ Food Standards Code Standard 2.5.3 Schedule 15 Schedule 18	Moderate
Japan (MHLW/CAA)	Functional‐food‐oriented regulatory framework (FOSHU/FFC)	Similar fermented milk standards; focus on functional benefits	Additives and ingredients regulated through positive lists and safety assessment systems	Highly developed health‐claim system, especially for gut health and lactose digestion	Food Sanitation Act Food Labeling Act FOSHU/FFC	Moderate
China (NHC/SAMR)	National food safety standards aligned increasingly with Codex Alimentarius	Requires *S. thermophilus* and *L. bulgaricus* as characteristic cultures	Positive‐list approach under GB 2760; stricter control for plain fermented milk	Health claims tightly regulated under national standards	GB 19302‐2025 GB 2760	Moderate

### 7.1 Regulations in the United States: The Food and Drug Administration (FDA) Regulatory Framework

In the United States, yogurt is a food governed by the US FDA under 21 CFR § 131.200, which was most recently amended by a 2021 Final Rule that consolidated the former separate standards for low‐fat and nonfat yogurt into a single unified standard (FDA 2023). Under this standard, yogurt is defined as the food produced by culturing one or more basic dairy ingredients with a bacterial culture containing *L*. *bulgaricus* and *S. thermophilus* and must contain not less than 3.25% milkfat (or be labeled accordingly if below this threshold) and not less than 8.25% milk solids, not including fat, with a pH of 4.6 or lower. It also allows the yogurt to be treated after culturing to inactivate viable microorganisms for extending shelf life. But if yogurt has been heat‐treated after culturing to inactivate viable microorganisms, the label must bear the phrase “does not contain live and active cultures.” Conversely, products containing ≥ 10^7^ CFU/g at manufacture with a reasonable expectation of ≥ 10^6^ CFU/g through shelf life may voluntarily claim “contains live and active cultures.”

Except for the obligate species *L. bulgaricus* and *S. thermophilus*, additional cultures including probiotic species such as *L. acidophilus*, *Bifidobacterium* spp., and *L. rhamnosus* may be added as optional ingredients under 21 CFR § 131.200(d)(1), provided they are either affirmed as GRAS under 21 CFR Part 184 or the subject of a GRAS notification accepted by FDA (FDA 2018). The FDA has a partial list of microorganisms and microbial‐derived ingredients used in food, so companies can submit GRAS Notices (GRNs) to the FDA's Office of Food Additive Safety for review on a voluntary basis. For instance, *Levilactobacillus brevis* LBR‐6108 (GRN No. 1229) and *L. rhamnosus* LGG (GRN No. 1013) have both undergone this process (Chr. Hansen Inc. 2021; Danisco USA Inc. 2024).

For the yogurt additives, optional ingredients allowed in yogurt according to 21 CFR § 131.200 are sweeteners, flavorings, color additives, stabilizers and emulsifiers, preservatives, and vitamins A and D, which must be either registered as artificial food substances under 21 CFR Part 170 through FDA pre‐approval or recognized as GRAS contents listed in 21 CFR Part 184 (Code of Federal Regulations 2004, 2016). Importantly, the FDA framework does not prescribe a static list of approved food additives for use in yogurt; rather, through the larger food additive and GRAS regulation system, manufacturers can utilize any substance considered “safe and suitable.” Likewise, other enzymes employed in yogurt production are also regulated as food additives or considered GRAS (e.g., lactase, transglutaminase, and chymosin). Several lactase preparations, including those produced from *K. lactis* and *Candida pseudotropicalis*, have been granted GRAS status and are used in dairy processing (Code of Federal Regulations 2016).

Overall, the US yogurt regulatory framework combines somewhat prescriptive product definitions with implementation routes that are relatively flexible. Although yogurt must by definition contain the two traditional starter cultures *S. thermophilus* and *L. bulgaricus*, and while US food law governing labeling and manufacture of yogurts is highly prescriptive with very strict requirements across various areas such as food safety, almost complete freedom in respect to many aspects concerning such things as additives, enzymes, or added probiotic cultures exists granted through GRAS and FDA's food additive systems (Susan 2023). The US system, in contrast to the more prescriptive positive‐list approaches adopted in areas such as the EU, is generally more industry‐led and flexible for yogurt product innovation.

### 7.2 Regulations in the EU: The EC/European Food and Safety Authority (EFSA) Regulatory Framework

The EU has one of the most holistic and prescriptive regulatory systems for food ingredients globally, which is based on a shared set of regulations adopted in December 2008, better known as the “Food Improvement Agents Package” (EU Specialty Food Ingredients 2018). For the yogurt section, the EU does not have a specific positive list for yogurt starter cultures; however, microbial safety is mainly assessed via the EFSA's Qualified Presumption of Safety (QPS) framework. Thus, well‐characterized microorganisms with established histories of safe use (e.g., *S. thermophilus*, *L. bulgaricus*, and other LAB) are regarded as safe for their intended usage, with no need to conduct a complete individual safety evaluation (EFSA 2007). EFSA is routinely revising its QPS framework, which forms the basis for an efficient regulatory risk assessment procedure of new probiotics and supplementary cultures contained in yogurt (EFSA Panel on Biological Hazards [BIOHAZ] et al. 2026). For live yogurt cultures, they may bear the health claim that “live cultures in yogurt or fermented milk improve lactose digestion in individuals who have difficulty digesting lactose.” To qualify for this claim, the product must contain at least $10^{8}$ CFU/g of live starter cultures, specifically *L. bulgaricus* and *S. thermophilus* (Kilara 2017).

For yogurt additives and enzymes, the EU adopts a highly centralized, precautionary regulatory framework. Under Regulations (EC) No 1333/2008 and 1332/2008, only additives explicitly authorized and included in the Union lists may be used while food enzymes are subject to the applicable authorization requirement. Following safety evaluation and demonstration of technological necessity (European Parliament 2008a, 2008b), additives are only permitted for specific product groups under defined conditions of use, like stabilizers, thickeners, acidity regulators, and other additives. Likewise, food enzymes must be undergo safety evaluation by EFSA; however, enzymes from QPS‐status microorganisms may benefit most from a streamlined screening process. The EU regulatory framework emphasizes consumer protection through harmonized legislation and strict premarket safety assessments. This has led to one of the world's strictest food regulatory systems today.

Both GRAS and the QPS system are intended to facilitate food safety by using pre‐existing scientific evidence; however, the two regulatory systems differ greatly in their implementation strategies and regulatory philosophies. Coverage of the concepts: US GRAS is broader and more flexible, allowing self‐affirmation under specific conditions, while the EU QPS concept is restricted to microorganisms and permits only centralized scientific assessment by EFSA prior to addition onto the QPS list.

### 7.3 Other Yogurt Regulations

The CAC, a joint body of the FAO and the WHO, establishes international food standards that serve as the reference point for the World Trade Organization's Agreement on Sanitary and Phytosanitary Measures (SPS Agreement; Alimentarius 2010). Codex Alimentarius is the international reference point for food. Codex Alimentarius has a similar definition for yogurt to that of the United States and the EU since Codex Alimentarius specifies that products fermented by *S. thermophilus* and *L. bulgaricus* with a minimum viable count on the date of minimum durability equal to or greater than 10^7^ CFU/g (CAC 2024). Regarding additives, Codex Alimentarius classifies fermented milk products into plain and flavored. In plain fermented milk, the use of preservatives, sweeteners, and emulsifiers is not technologically justified, making the Codex Alimentarius framework more closely aligned with the restrictive EU approach than with the comparatively permissive US regulatory system. Nevertheless, unlike the EU regulatory framework, which has established a harmonized authorization system for food enzymes, Codex Alimentarius does not provide a dedicated positive list for approved food enzymes, and the regulation of enzyme use therefore remains largely subject to national legislation (Sutay Kocabaş and Grumet 2019).

Japan's food products are under the regulation of the Ministry of Health, Labour, and Welfare (MHLW) and Consumer Affairs Agency (CAA; Freyr 2026). Japan adopts a distinct regulatory strategy that emphasizes functional foods and health claims. In addition to general food safety regulations, yogurt products may fall under specialized categories such as Foods for Specified Health Uses (FOSHU) and Foods with Function Claims (FFC), which require scientific evidence supporting claimed physiological benefits (Kamioka et al. 2019). Yogurt manufacture in Japan, as in other regions, mainly involves *S. thermophilus* and *L. bulgaricus*, and novel food applications of additives and enzyme preparations are controlled by positive lists or safety assessments. In contrast, Japan's framework focuses more on validating probiotic functionality, gut health, and digestive claims for fermented dairy products (Mukherjee et al. 2022).

In China, yogurt and fermented milk products are regulated by the National Health Commission (NHC) and the State Administration for Market Regulation (SAMR). The current regulatory framework has increasingly aligned with Codex standards through GB 19302‐2025 National Food Safety Standard Fermented Milk and GB 2760 for food additives, especially mandatory starter cultures and category‐based additive management (National Health Commission of PRC 2024, 2025). Like the EU, China uses a strict positive list, and only authorized additives and enzymes can be used. In contrast, Australia and New Zealand apply a Codex‐consistent composition for yogurt but maintain a more flexible risk‐based framework through the Food Standard Australia New Zealand (FSANZ; FSANZ 2026). Although additives and enzymes are still regulated through positive lists and safety assessment, the system allows case‐by‐case applications and public consultation for new preservative uses, such as natamycin for mold control in yogurt (DSM Food Specialties Australia Pty Ltd 2014).

To sum up, we predict that clear and harmonized regulatory frameworks of starter cultures, enzyme applications, and clean‐label requirements as well as GMO‐related labeling will become increasingly necessary to facilitate industrial applications and international trade between yogurt producers. Reducing regulatory uncertainty and leveraging health benefits can improve the climate for yogurt innovation by increasing transparency and better coordination among national government authorities, starter culture suppliers, and dairy producers. Longer‐term advancements in microbial safety assessment (such as metagenomics), precision fermentation, and functional‐food regulation could enable smoother global regulatory pathways that incorporate new yogurt technologies such as novel starter cultures, advanced bioprotective systems, and new probiotic functional products.

## 8 Conclusion

Current strategies for producing yogurt are increasingly founded on biological and clean‐label‐integrated solutions that can enhance yogurt stability, shelf life, nutritional functionality, and lower the overall cost of production. Innovations such as optimization of starter cultures, bioprotective systems, and lactose‐reduction strategies offer promising pathways to improve quality and meet consumer demand for natural yogurt products. In terms of reducing costs, an obvious target is the starter culture, which accounts for as much as 10% of the price of raw milk. Here, exploring the use of bulk starters offers a tangible opportunity to improve economic efficiency without compromising quality. There are, however, hurdles associated with the transition to more sustainable yogurt production, for example, the gap between research and implementation of research results; not only for dairies that often are bound by tradition but also for the culture providers that frequently lag behind in terms of adopting innovative solutions. In addition, there can be regulatory issues among countries that need to be dealt with, the economic viability of novel solutions that need to be assessed prior to industrial adoption, as well as consumer acceptance. To facilitate the introduction of innovation to the market, close cooperation among dairy manufacturers, starter‐culture suppliers, enzyme and ingredient producers, regulatory authorities, and researchers is essential.

## Author Contributions

**Shuangqing Zhao**: conceptualization, methodology, writing – original draft, writing – review and editing, visualization, software, investigation. **Belay Tilahun Tadesse**: methodology, writing – review and editing. **Christian Solem**: conceptualization, writing – original draft, writing – review and editing, supervision, funding acquisition, project administration.

## Conflicts of Interest

The authors declare no conflicts of interest.
